# Supplementary material for: Machine learning approaches reveal genomic regions associated with sugarcane brown rust resistance
Source: Sci Rep. 2020 Nov 18;10:20057. doi: 10.1038/s41598-020-77063-5 (PMC7676261; doi:10.1038/s41598-020-77063-5)
Supplement: Supplementary file 1 — Supplementary Information. [file 41598_2020_77063_MOESM1_ESM.pdf]

SUPPLEMENTARY INFORMATION

Machine learning approaches reveal genomic regions associated with sugarcane brown rust resistance

Alexandre Hild Aono, Estela Araujo Costa, Hugo Vianna Silva Rody, James Shiniti Nagai, Ricardo José Gonzaga Pimenta, Melina Cristina Mancini, Fernanda Raquel Camilo dos Santos, Luciana Rossini Pinto, Marcos Guimarães de Andrade Landell, Anete Pereira de Souza, and Reginaldo Massanobu Kuroshu

1. Population Organization

The biparental population included: (1) the parents IACSP95-3018 and IACSP93-3046, named 3018 and 3046, respectively; (2) the controls SP81-3250 and RB835486, named 3250 and 5486, respectively; and (3) the progeny composed of 219 individuals, numbered from 1 to 220 (with the exception of 57, which died during the experiment). Genotypic data were obtained for 180 individuals, named: 3, 5, 7, 8, 13, 14, 15, 18, 20, 21, 23, 24, 25, 26, 27, 29, 30, 33, 34, 35, 36, 37, 38, 39, 40, 41, 42, 43, 44, 45, 46, 47, 50, 51, 52, 53, 54, 55, 56, 58, 61, 63, 64, 65, 66, 67, 68, 69, 70, 71, 72, 73, 74, 76, 77, 78, 79, 80, 81, 84, 85, 86, 87, 88, 89, 90, 91, 92, 93, 94, 95, 96, 97, 98, 99, 100, 101, 102, 103, 104, 105, 106, 107, 108, 109, 110, 112, 113, 114, 115, 116, 117, 118, 119, 120, 121, 122, 123, 124, 125, 127, 128, 129, 130, 131, 132, 133, 134, 135, 136, 137, 139, 140, 141, 142, 143, 144, 145, 146, 147, 148, 150, 151, 152, 153, 154, 155, 156, 157, 158, 159, 160, 161, 162, 163, 164, 165, 166, 167, 168, 170, 171, 172, 174, 175, 176, 177, 180, 181, 182, 183, 184, 185, 186, 187, 188, 189, 190, 191, 192, 193, 194, 195, 196, 198, 200, 201, 202, 203, 205, 206, 209, 210, 211, 212, 214, 215, 217, 218, and 220.

2. Phenotypic Analyses

Using the original brown rust phenotypes and their normalized values for all the available individuals, we checked the residual distribution based on the created mixed model. With the Shapiro-Wilk test, we obtained

I. A p-value of 0.00000000000001160501 for the built model based on the original values and

II. A p-value of 0.07729799 for the built model based on the normalized values.

It is clear that the original dataset does not follow a normal distribution, in contrast to the normalized data. As shown in Supplementary Fig. S1, the same conclusion can be reached by visualizing the Q-Q plots. Therefore, we decided to perform our analyses using the dataset of normalized values. With these values, we estimated the best linear unbiased prediction (BLUP) values for the genotyped individuals. Using these values, we constructed a pairwise Euclidean distance matrix and performed complete hierarchical clustering to check for patterns in the dataset. There was clear separation of the 2 groups in the dendrogram shown in Supplementary Fig. S2.

However, to properly define these two groups, we used two different approaches: (1) the within-cluster sum of squares for different numbers of defined clusters and (2) the average silhouette width of clusters with different numbers of groups. The results are shown in Supplementary Figs. S3 and S4. For both methods, the most appropriate number of clusters was two, as expected.

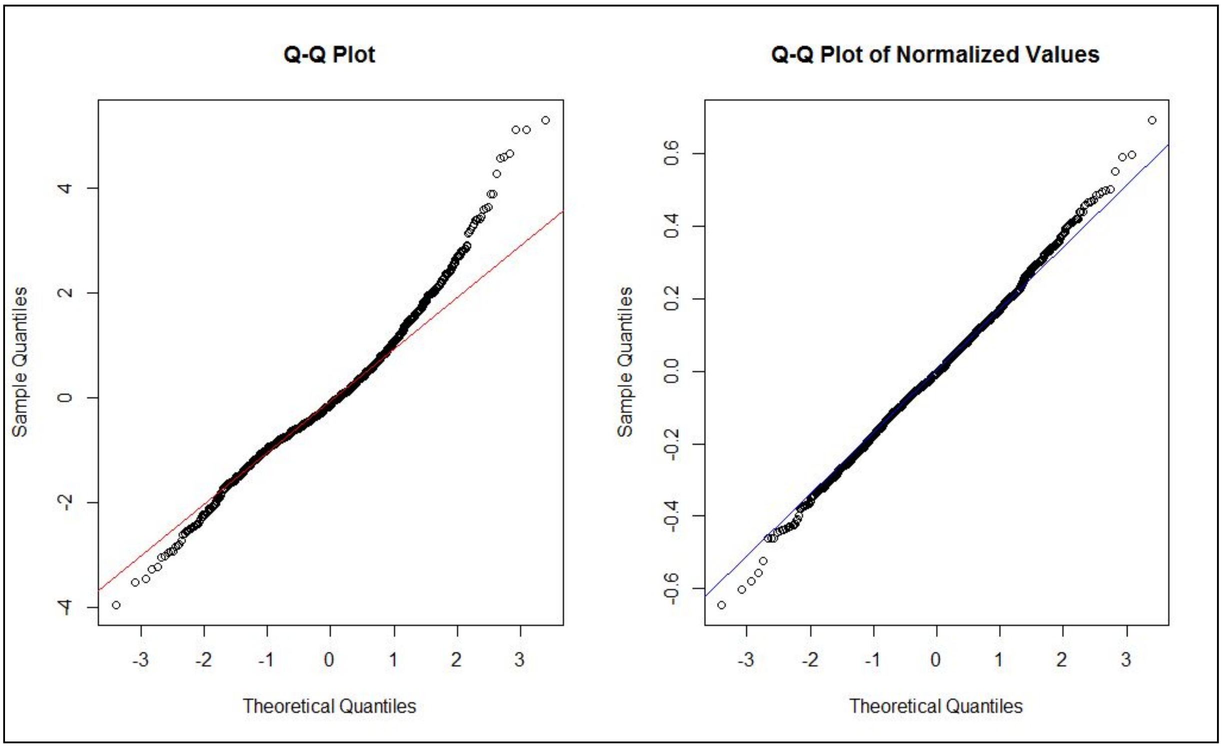

Supplementary Fig. S1. Quantile-quantile (Q-Q) plots of brown rust residual values for the estimated mixed model.

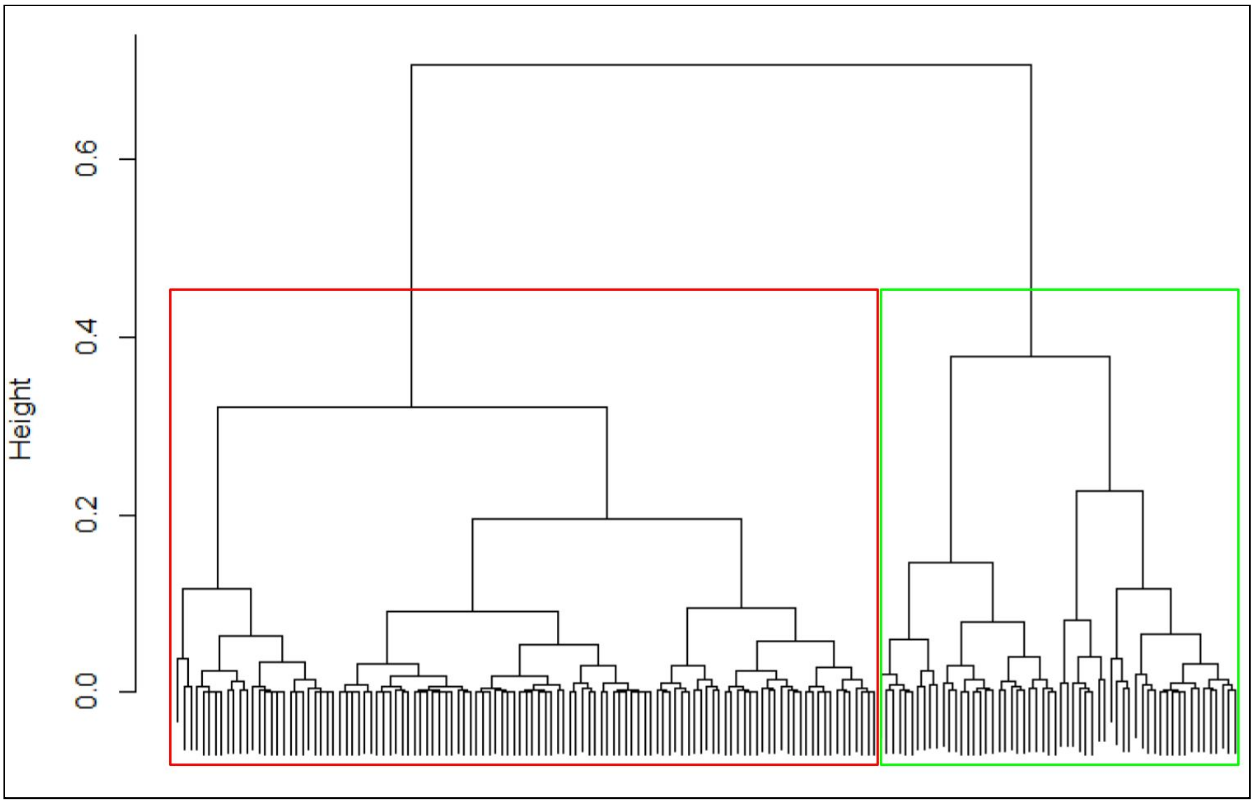

Supplementary Fig. S2. Dendrogram based on the hierarchical clustering of Euclidean distances calculated with best linear unbiased prediction (BLUP) values obtained through the estimated brown rust mixed model.

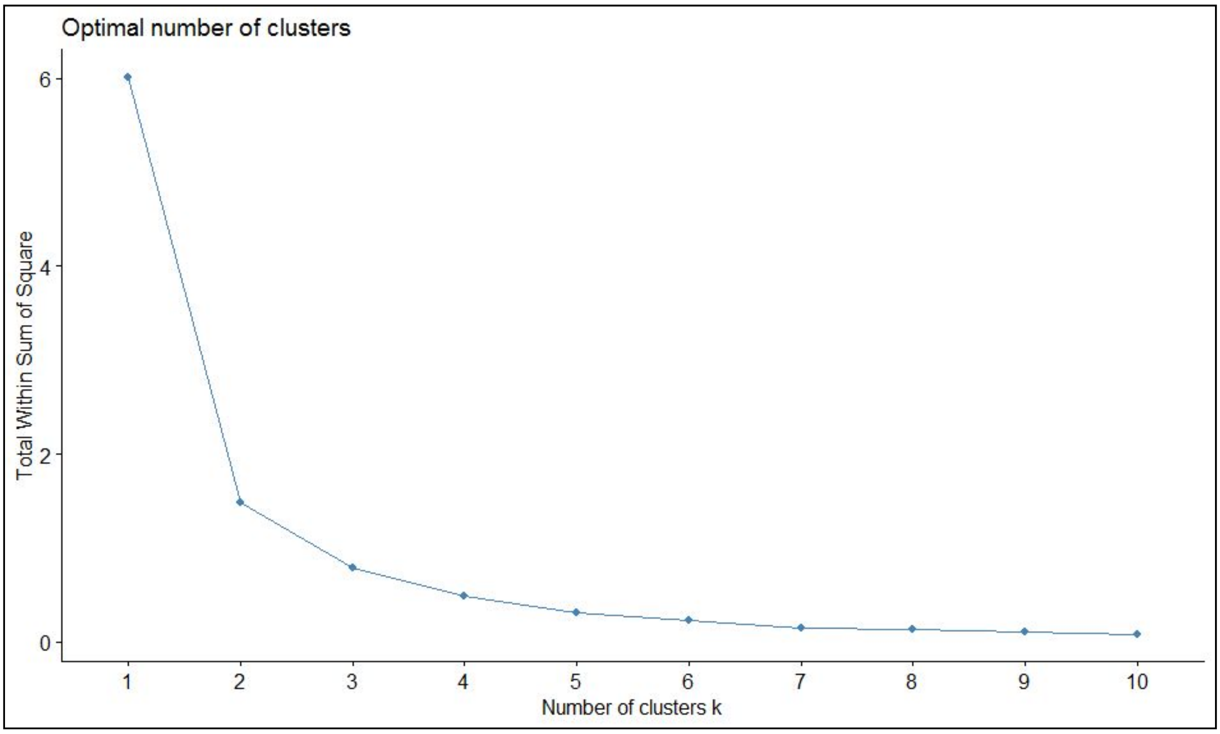

Supplementary Fig. S3. Evaluation of the ideal quantity of clusters on the basis of within-cluster sums of squares and the best linear unbiased prediction (BLUP) values.

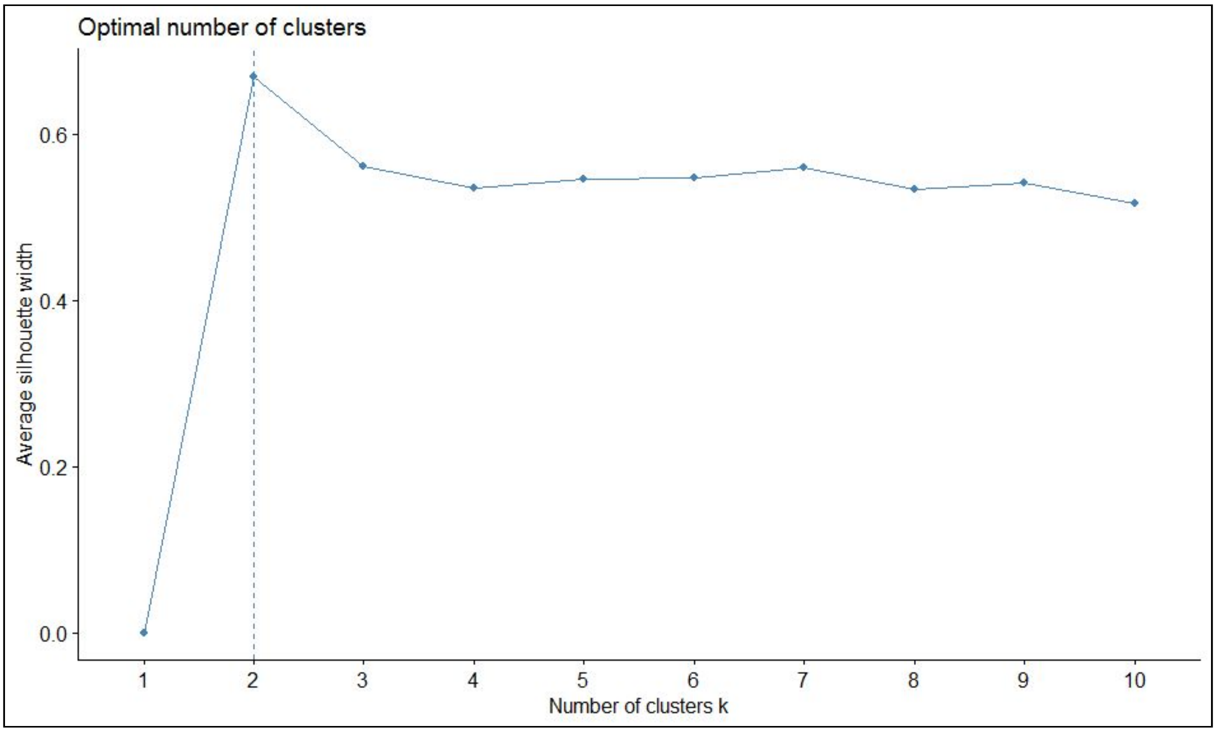

Supplementary Fig. S4. Evaluation of the ideal quantity of clusters on the basis of average silhouette width and the best linear unbiased prediction (BLUP) values.

The difference in BLUP values was highly variable, as shown in Supplementary Fig. S5. When evaluating all the original values without considering the BLUPs, clear differentiation was also observed (Supplementary Fig. S6), with only a few outliers. These discrepant values can be explained by the presence of nongenetic effects on the collected phenotypes. By performing a T-test between the values of these samples, we obtained a p-value of approximately 0.00000000000000022, showing significant divergence between the means.

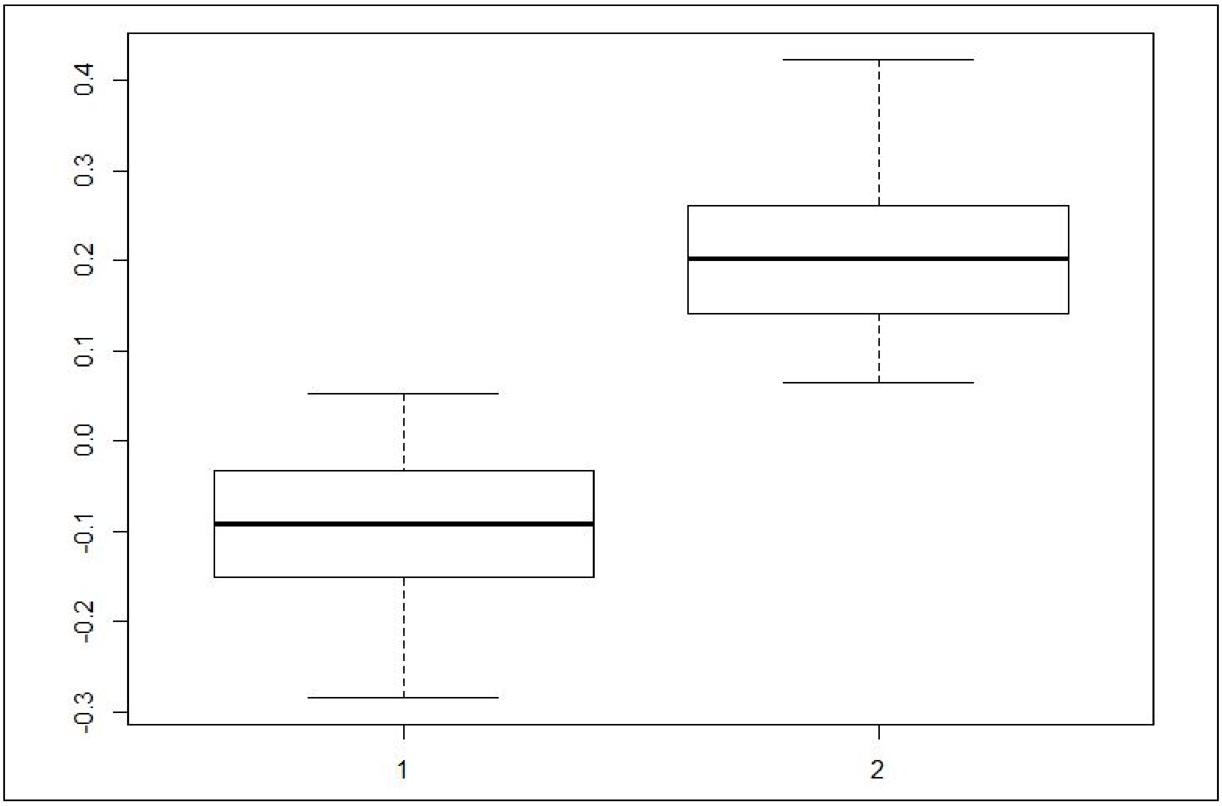

Supplementary Fig. S5. Boxplots representing the differences in best linear unbiased prediction (BLUP) values between the identified phenotypic groups through BLUP clustering analyses.

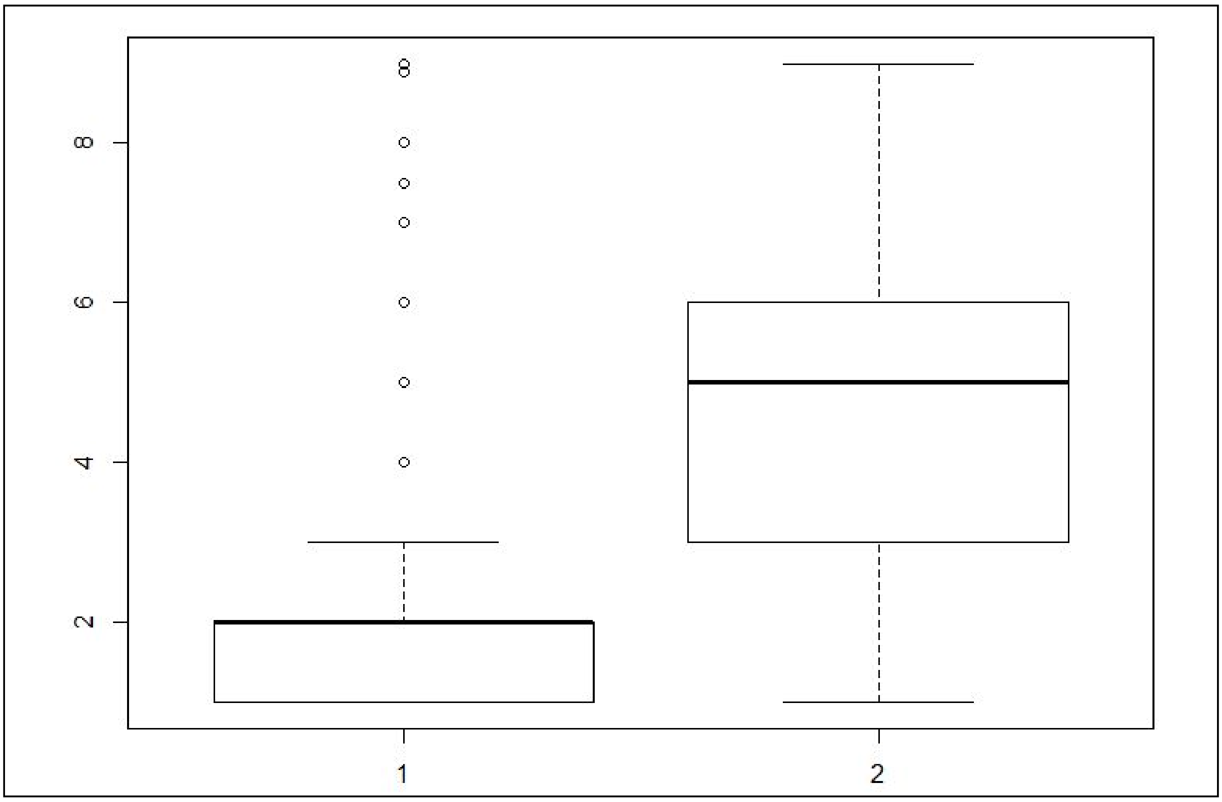

Supplementary Fig. S6. Boxplots representing the differences in phenotypic values between the identified groups through best linear unbiased prediction (BLUP) clustering analyses.

Given these findings, we continued our analyses using these two groups instead of the evaluation scores due to the clearly different susceptibility profiles. Therefore, we considered the individuals as belonging to group 1, named resistant, or group 2, named susceptible.

3. Quality Control of Sequencing Data

Using the established quality filters on raw sequencing data, we kept 75.68% of the data (Supplementary Table S1). These data represent a subset of the original data with high-quality metrics, especially considering the demultiplexing process where there were no significant losses (Supplementary Table S2).

Supplementary Table S1. Quantity of reads in the different sequencing flow cells with and without quality filtering using the built genotyping by sequencing (GBS) libraries. The sequencing experiments were performed in 2015 and 2017 (2 times), separating the sugarcane population into two groups (Group 1 and Group 2).

	Raw Reads	Filtered Reads
GBS 2015	164,171,329	134,809,753 (82.12%)

GBS 2017 - Group 1 (1st time)	149,635,834	85,653,323 (57.24%)
GBS 2017 - Group 2 (1st time)	341,969,762	278,490,414 (81.44%)
GBS 2017 - Group 2 (2nd time)	311,792,539	257,685,281 (82.65%)
GBS 2017 - Group 1 (2nd time)	135,593,786	78,270,511 (57.72%)
**Total**	**1,103,163,250**	**834,909,282 (75.68%)**

Supplementary Table S2. Quantity of reads kept after demultiplexing steps. The genotyping by sequencing (GBS) experiments were performed in 2015 and 2017 (2 times), separating the sugarcane population into two groups (Group 1 and Group 2).

	**Reads with Barcode Correspondence**	**Reads without Barcode Correspondence**
GBS 2015	134,809,665	88
GBS 2017 - Group 1 (1st time)	85,652,218	1,105
GBS 2017 - Group 2 (1st time)	278,489,562	852
GBS 2017 - Group 2 (2nd time)	257,684,834	447
GBS 2017 - Group 1 (2nd time)	78,270,085	426
**Total**	**834,906,364**	**2,918**

4. Read Alignment

The filtered read alignments against the seven selected references and the 3 adjacent subsets of the *S. spontaneum* genome obtained using BWA and Bowtie2 presented discrepancies in alignment quantity. Only uniquely mapped reads were kept for single nucleotide polymorphism (SNP) calling, and the BWA results included more of these reads than did the Bowtie2 results (40.23% compared to 34.65%), presented in Supplementary Table S3. In addition, based on the total number of aligned reads, the percentage of loss was smaller for the BWA alignments than for the Bowtie2 alignments. The mean percentage of read maintenance was approximately 10% larger with BWA than with Bowtie2. Due to this more efficient use of our data, we selected BWA alignments for SNP detection.

Supplementary Table S3. Analysis of mapped reads using BWA and Bowtie2 software against the following genomic references: the methyl-filtered (MF) genome of sugarcane cultivar SP70-1143, the sorghum genome from Phytozome v.13, a sugarcane leaf transcriptome (RNASeq), the draft genome of the hybrid SP80-3280, the monoploid genome of the R570 variety, the *Saccharum spontaneum* (SS) genome split into four subsets on the basis of

the allele-defined genome (A1, A2, A3, and A4), and sequences from the sugarcane expressed sequence tag project (Sucest).

	**BWA Mapped Reads**	**BWA Uniquely Mapped Reads**	**Percentage of Maintenanc e**	**Bowtie Mapped Reads**	**Bowtie Uniquely Mapped Reads**	**Percentage of Maintenanc e**
MF genome	787,597,773 (94.33%)	580,694,803 (69.55%)	73.73%	790,307,868 (94.66%)	415,316,587 (49.74%)	52.55%
Sorghum	372,476,439 (44.61%)	334,543,933 (40.07%)	89.82%	379,731,080 (45.48%)	312,688,984 (37.45%)	82.34%
SP80-3280	612,219,802 (73.33%)	256,830,971 (30.76%)	41.95%	626,857,213 (78.08%)	203,710,582 (24.40%)	32.50%
R570	453,755,780 (54.35%)	371,364,888 (44.48%)	81.84%	465,348,929 (55.74%)	339,658,623 (40.68%)	73.00%
RNASeq	286,463,784 (34.31%)	282,308,600 (33.81%)	98.55%	295,867,783 (35.44%)	279,018,734 (33.42%)	94.31%
Sucest	313,409,778 (37.54%)	165,927,835 (19.87%)	52.94%	323,237,607 (38.72%)	155,002,063 (18.57%)	47.95%
SS A1	595,267,463 (71.30%)	342,151,259 (41.00%)	57.48%	609,900,489 (73.05%)	298,041,048 (35.70%)	48.87%
SS A2	594,220,509 (71.17%)	346,818,994 (41.54%)	58.37%	609,010,287 (72.94%)	301,122,972 (36.07%)	49.44%
SS A3	576,398,435 (69.04%)	336,411,572 (40.30%)	58.37%	590,092,154 (70.68%)	294,076,883 (35.22%)	49.84%
SS A4	587,266,198 (70.34%)	341,480,330 (40.90%)	58.15%	599,587,919 (71.81%)	293,878,819 (35.20%)	49.01%
**Mean**	**62.03%**	**40.23%**	**67.12%**	**63.66%**	**34.65%**	**57.98%**

Supplementary Fig. S7 provides an overview of the alignment process across the different references. Due to very shallow sequencing depths, individuals 33, 39, 41, 47, 51, 56, 68, 85, 87, 93 and 107 were not considered in further analyses (Supplementary Fig. S7-b). Either on different scales (original and logarithmic) or with different references, the discrepancy in these observations when compared to the entire dataset was evident, and bias in further analyses could potentially be introduced if using these individuals, such as filtering SNP positions due to the small quantity of individuals.

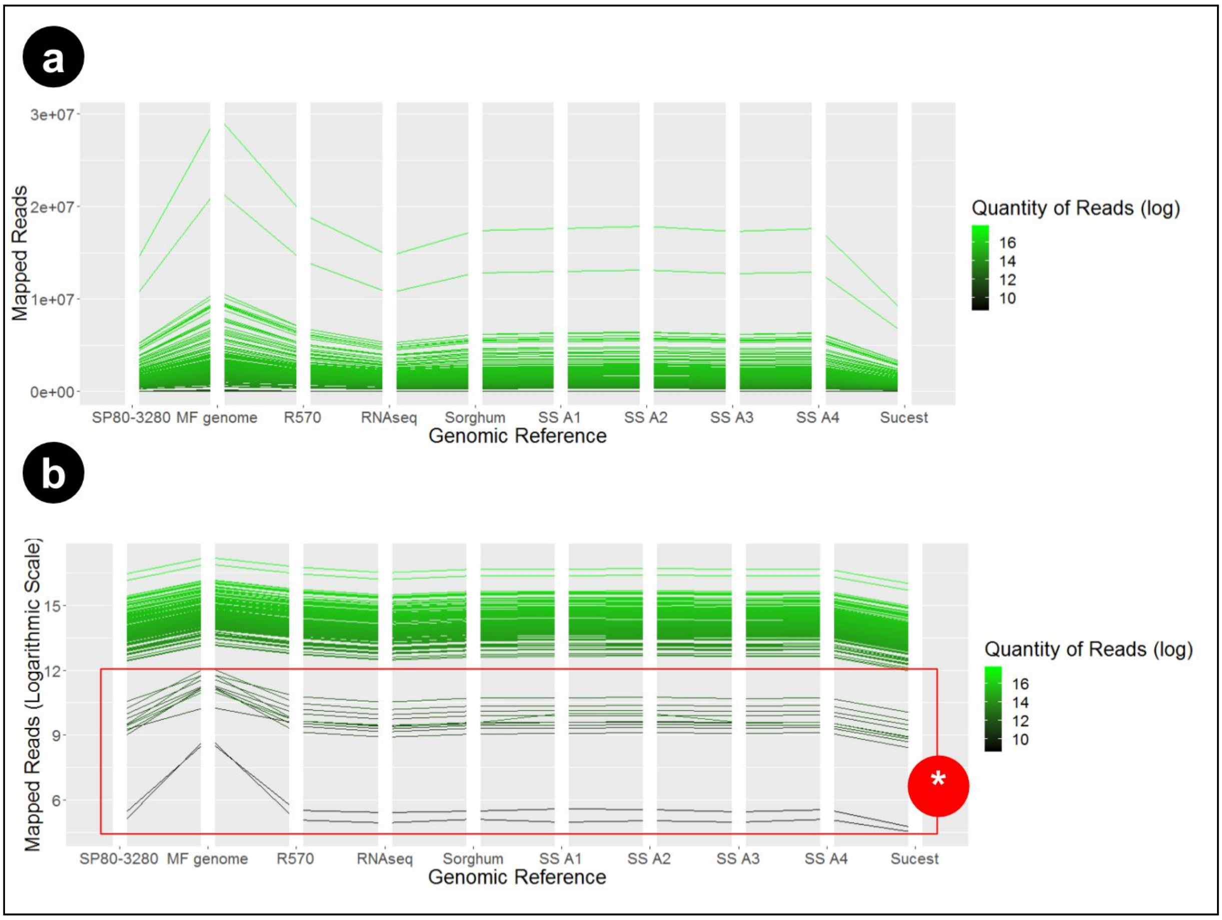

Supplementary Fig. S7. Analysis of mapped reads against the methyl-filtered (MF) genome of sugarcane cultivar SP70-1143, the sorghum genome from Phytozome v.13, a sugarcane leaf transcriptome (RNASeq), the draft genome of the hybrid SP80-3280, the monoploid genome of the R570 variety, the *Saccharum spontaneum* (SS) genome split into four subsets on the basis of the allele-defined genome (A1, A2, A3, and A4), and sequences from the sugarcane expressed sequence tag project (Sucest). The lines in the plots are colored based on the quantity of reads per individual and presented in two schemes: (a) the raw quantity of mapped reads and (b) the quantity of mapped reads on a logarithmic scale. Individuals removed from the analyses are represented by (*).

5. Reference Evaluation

To evaluate the reads' correspondence to the different references used, we compared the results shown in Supplementary Tables S4 and S5. Although we had references containing more information than the MF genome (Supplementary Table S4), we obtained more extensive alignments of our data to the MF reference (Supplementary Table S5), which was built according to the obtained consensus contigs. The total length of MF contigs was at least 6.5 times greater than that of the other references, and the number of contigs was at least 6.6 times higher, indicating a larger percentage of genomic regions with similarity to the GBS data. Although the N50 metric for the original MF scaffolds was lower than that for the other references,

the value had another interpretation when using the consensus sequences, where all the regions had similar values. However, analysis of the L50 metric together with the total quantity of contigs corroborated that the MF alignments produced longer sequences.

Supplementary Table S4. Analysis of different reference compositions based on the total number of base pairs (bp), the quantity of Ns per 100 kbp, the number of contigs/scaffolds, the largest sequence, and the metric N50. The used references were the methyl-filtered (MF) genome of sugarcane cultivar SP70-1143, the sorghum genome from Phytozome v.13, a sugarcane leaf transcriptome (RNASeq), the draft genome of the hybrid SP80-3280, the monoploid genome of the R570 variety, the *Saccharum spontaneum* (SS) genome split into four subsets on the basis of the allele-defined genome (A1, A2, A3, and A4), and sequences from the sugarcane expressed sequence tag project (Sucest).

	**Total (bp)**	**Ns per 100 kbp**	**No. Contigs or Scaffolds**	**Largest Contig (bp)**	**N50 (bp)**
MF genome	674,032,540	46,984.06	1,109,444	35,917	2,083
Sorghum	708,863,705	4,720.34	870	80,884,392	68,658,214
SP80-3280	1,169,948,913	0.00	199,028	115,913	8,451
R570	426,798,785	8,814.84	211	72,286,729	45,576,573
RNAseq	66,572,642	0.00	72,269	17,055	1,737
Sucest	133,946,191	0.00	195,765	8,854	1,258
SS A1	974,684,959	198.19	15,744	121,903,222	79,322,609
SS A2	984,574,931	198.97	15,744	123,268,843	90,128,929
SS A3	963,861,475	197.93	15,744	126,636,275	89,762,077
SS A4	938,617,136	194.65	15,744	116,847,860	84,617,075

Supplementary Table S5. Consensus sequence profiles obtained via read mapping against the different references based on the quantity of contigs, the total number of base pairs (bp), the number of Ns per 100 kbp, the largest sequence, and the metrics N50, N75, L50, and L75. The used references were the methyl-filtered (MF) genome of sugarcane cultivar SP70-1143, the sorghum genome from Phytozome v.13, a sugarcane leaf transcriptome (RNASeq), the draft genome of the hybrid SP80-3280, the monoploid genome of the R570 variety, the *Saccharum spontaneum* (SS) genome split into four subsets on the basis of the allele-defined genome (A1, A2, A3, and A4), and sequences from the sugarcane expressed sequence tag project (Sucest).

	**Contigs**	**Total (bp)**	**Ns per 100 kbp**	**Largest Contig (bp)**	**N50/N75**	**L50/L75**

MF genome	82,472	7,087,591	60.51	120	86/86	40,353/ 60,957
Sorghum	35,174	3,025,392	474.38	123	86/86	17,058/ 25,852
SP80-3280	43,639	3,759,046	80.66	123	86/86	21,414/ 32,342
R570	48,663	4,203,728	92.13	118	86/86	23,829/ 36,049
RNAseq	25,673	2,205,254	73.01	116	86/86	12,536/ 18,946
Sucest	15,335	1,311,672	77.76	120	86/86	7,466/ 11,279
SS A1	49,487	4,271,185	131.44	124	86/86	24,185/ 36,602
SS A2	49,616	4,281,974	126.95	121	86/86	24,239/ 36,687
SS A3	47,994	4,142,664	130.23	122	86/86	23,453/ 35,496
SS A4	48,600	4,195,855	138.90	121	86/86	23,744/ 35,941

It might be intuitive to expect that with more extensive alignments (i.e., greater coverage), the sequencing depths across MF scaffolds would be lower. However, this was not observed. MF alignments produced a larger number of genomic positions with high correspondence than did other alignments (Supplementary Table S5). However, as shown in Supplementary Table S6, these positions also had increased depth. With at least 44.38% more coverage than the other references, MF alignments had a larger quantity of reads corroborating genomic positions. Considering a minimum depth of 8.000 reads, MF had at least 64.50% more covered regions than the other references.

Supplementary Table S6. Depth mapping profiles of different references and the genotyping by sequencing (GBS) data. The used references were the methyl-filtered (MF) genome of sugarcane cultivar SP70-1143, the sorghum genome from Phytozome v.13, a sugarcane leaf transcriptome (RNASeq), the draft genome of the hybrid SP80-3280, the monoploid genome of the R570 variety, the *Saccharum spontaneum* (SS) genome split into four subsets on the basis of the allele-defined genome (A1, A2, A3, and A4), and sequences from the sugarcane expressed sequence tag project (Sucest).

	Total Positions	Total Positions where Mapping Depth > Value					
		>=100	>=500	>=1000	>=5000	>=6000	>=8000
MF genome	13,694,206	5,630,873	3,855,721	3,060,666	1,343,310	1,172,174	739,799

Sorghum	6,577,944	2,750,262	1,961,559	1,594,831	773,279	683,861	438,040
SP80-3280	8,119,350	2,881,841	1,915,561	1,480,226	615,468	536,882	336,060
R570	8,300,203	3,236,343	2,234,123	1,788,540	822,918	722,547	449,732
RNAseq	4,482,524	1,978,380	1,414,520	1,150,812	558,839	494,594	317,338
Sucest	2,857,978	1,248,375	886,462	719,308	342,231	305,128	199,139
SS A1	9,464,530	3,339,323	2,256,387	1,778,348	789,632	690,830	427,588
SS A2	9,485,051	3,358,500	2,282,746	1,810,360	812,133	708,668	436,856
SS A3	9,254,628	3,243,610	2,197,171	1,740,758	776,469	680,257	421,163
SS A4	9,282,252	3,273,861	2,221,450	1,754,522	782,224	685,346	425,154

The MF sugarcane genome had the largest number of total mapped positions. Additionally, this reference also had the largest number of mapped positions in five of the six evaluated depth ranges. Importantly, in addition to having the greatest observed depths, these reads were better distributed among the scaffolds, as shown by the quantity of mapped positions. On the basis of these conclusions, we evaluated redundancy among the consensus sequences obtained by the mapping process against the different references. Supplementary Table S7 and Supplementary Fig. S8 show this redundancy. MF contigs had the largest values of correspondence, i.e., they included the majority of genomic information contained in all other references.

Supplementary Table S7. Consensus sequence redundancies across different references assessed via comparative alignments (rows represent the queries, and the columns, the targets). The used references were the methyl-filtered (MF) genome of sugarcane cultivar SP70-1143, the sorghum genome from Phytozome v.13, a sugarcane leaf transcriptome (RNASeq), the draft genome of the hybrid SP80-3280, the monoploid genome of the R570 variety, the *Saccharum spontaneum* (SS) genome split into four subsets on the basis of the allele-defined genome (A1, A2, A3, and A4), and sequences from the sugarcane expressed sequence tag project (Sucest).

	MF genome	Sorghum	SP80-3280	R570	RNAseq	Sucest	SS A1	SS A2	SS A3	SS A4
MF genome	82,472 (100%)	25,072 (71.28%)	27,733 (78.37%)	35,249 (72.43%)	20,093 (78.37%)	11,670 (76.10%)	33,509 (67.71%)	33,925 (68.38%)	32,650 (68.03%)	33,087 (68.08%)
Sorghum	24,647 (29.89%)	35,174 (100%)	10,124 (23.20%)	16,963 (34.86%)	11,927 (46.46%)	6,613 (43.13%)	14,792 (29.90%)	15,087 (30.41%)	14,634 (30.49%)	14,700 (30.25%)
SP80-3280	27,825 (33.74%)	10,340 (29.40%)	43,639 (100%)	16,418 (33.74%)	8,935 (34.80%)	5,216 (34.01%)	16,474 (33.29%)	16,556 (33.37%)	15,792 (32.90%)	15,989 (32.90%)
R570	35,078 (42.53%)	17,163 (48.80%)	16,206 (37.14%)	48,663 (100%)	12,650 (49.27%)	7,247 (47.26%)	21,321 (43.08%)	21,362 (43.05%)	21,135 (44.04%)	21,319 (43.87%)
RNAseq	19,634 (23.81%)	11,851 (33.69%)	8,703 (19.94%)	12,426 (25.53%)	25,673 (100%)	8,328 (54.31%)	11,115 (22.46%)	11,199 (22.57%)	10,902 (22.72%)	11,148 (22.94%)
Sucest	11,380 (13.80%)	6,590 (18.74%)	5,068 (11.61%)	7,115 (14.62%)	8,325 (32.43%)	15,335 (100%)	6,623 (13.38%)	6,601 (13.30%)	6,324 (13.18%)	6,542 (13.46%)

SS A1	33,196 (40.25%)	14,940 (42.47%)	16,196 (37.11%)	21,255 (43.68%)	11,260 (43.86%)	6,729 (43.88%)	49,487 (100%)	25,256 (50.90%)	24,370 (50.78%)	24,837 (51.10%)
SS A2	33,596 (40.74%)	15,199 (43.21%)	16,235 (37.20%)	21,306 (43.78%)	11,368 (44.28%)	6,690 (43.63%)	25,227 (50.98%)	49,616 (100%)	24,599 (51.25%)	24,905 (51.24%)
SS A3	32,321 (39.19%)	14,731 (41.88%)	15,526 (35.58%)	21,092 (43.34%)	11,049 (43.04%)	6,410 (41.80%)	24,330 (49.16%)	24,612 (49.60%)	47,994 (100%)	23,789 (48.95%)
SS A4	32,797 (39.77%)	14,837 (42.18%)	15,697 (35.97%)	21,286 (43.74%)	11,316 (44.08%)	6,654 (43.39%)	24,866 (50.25%)	24,918 (50.22%)	23,855 (49.70%)	48,600 (100%)

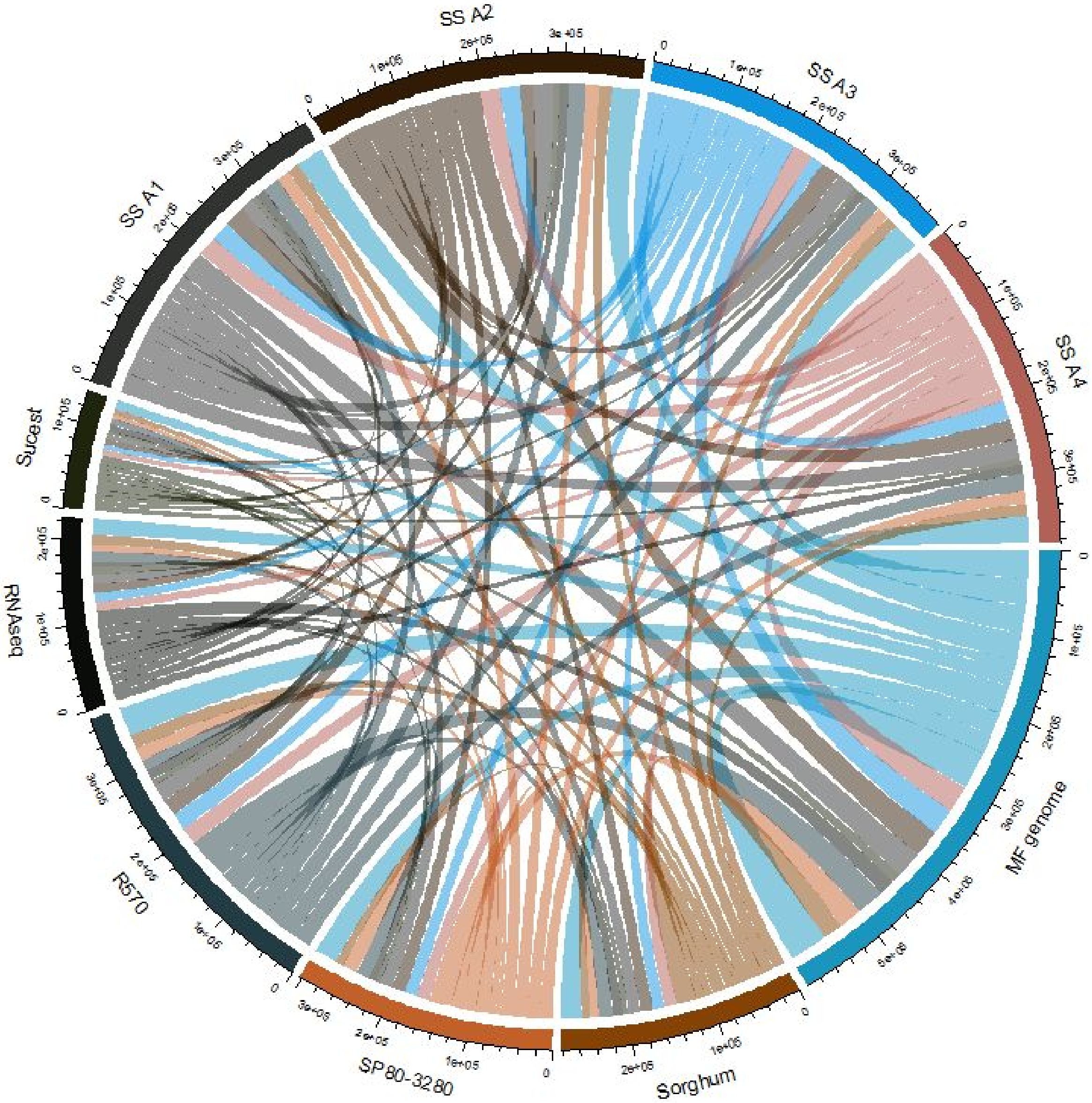

Supplementary Fig. S8. Redundancies of consensus sequences visualized in a Circos plot. The used references were the methyl-filtered (MF) genome of sugarcane cultivar SP70-1143, the sorghum genome from Phytozome v.13, a sugarcane leaf transcriptome (RNASeq), the draft genome of the hybrid SP80-3280, the monoploid genome of the R570 variety, the *Saccharum spontaneum* (SS) genome split into four subsets on the basis of the allele-defined genome (A1, A2, A3, and A4), and sequences from the sugarcane expressed sequence tag project (Sucest).

As a final step in considering the MF genome as our most appropriate option for the analyses, we evaluated the quantity of SNPs identified by Tassel and Stacks, tools developed to identify these variants in GBS and RADSeq data. Due to the specificity of these experiments in selecting genomic regions, these tools were selected as another reference evaluation parameter. As shown in Supplementary Table S8, raw variants called by Tassel and the MF reference genome allowed the identification of at least 51% more variants than did those obtained with the other references.

Supplementary Table S8. Different variants identified by Tassel using as references the methyl-filtered (MF) genome of sugarcane cultivar SP70-1143, the sorghum genome from Phytozome v.13, a sugarcane leaf transcriptome (RNASeq), the draft genome of the hybrid SP80-3280, the monoploid genome of the R570 variety, the *Saccharum spontaneum* (SS) genome split into four subsets on the basis of the allele-defined genome (A1, A2, A3, and A4), and sequences from the sugarcane expressed sequence tag project (Sucest).

	Raw Variants	Biallelic SNPs	Multiallelic SNPs	Indels
MF genome	137,757	135,594	385	1,778
Sorghum	63,653	62,467	105	1,081
SP80-3280	63,625	62,781	208	636
R570	91,108	89,795	147	1,166
RNAseq	60,214	59,291	57	866
Sucest	38,036	37,363	70	603
SS A1	86,634	85,419	171	1,044
SS A2	89,038	87,728	225	1,085
SS A3	85,306	84,119	157	1,030
SS A4	86,563	85,358	176	1,029

The same result was observed when using Stacks (Supplementary Table S9). The number of identified variants was much larger when using the MF reference than when using the other references, even when using the established filters.

Supplementary Table S9. Identification of SNPs using Stacks and Tassel and stringent filters (at least 50 reads per individual and a maximum of 25% missing data) and as references the methyl-filtered (MF) genome of sugarcane cultivar SP70-1143, the sorghum genome from Phytozome v.13, a sugarcane leaf transcriptome (RNASeq), the draft genome of the hybrid SP80-3280, the

monoploid genome of the R570 variety, the *Saccharum spontaneum* (SS) genome split into four subsets on the basis of the allele-defined genome (A1, A2, A3, and A4), and sequences from the sugarcane expressed sequence tag project (Sucest).

	# of Tassel Biallelic SNPs	# of Tassel Filtered Biallelic SNPs	# of Stacks Biallelic SNPs	# of Stacks Filtered Biallelic SNPs	# Intersecting Biallelic SNPs
MF genome	137,757	18,881	106,881	2,605	50,274
Sorghum	63,653	10,262	44,074	1,544	16,159
SP80-3280	63,624	7,552	56,830	1,033	25,162
R570	91,108	13,441	72,378	1,784	33,752
RNAseq	60,214	10,881	38,641	1,296	18,715
Sucest	38,036	6,422	23,545	623	11,001
SS A1	86,634	11,536	72,638	1,518	32,570
SS A2	89,038	11,763	73,536	1,600	33,098
SS A3	85,306	11,726	70,938	1,492	31,612
SS A4	86,563	11,562	71,539	1,502	31,876

6. SNP Calling

SNP calling was also performed with GATK, FreeBayes and SAMtools. The tools produced different quantities of identified markers, confirming the influence of the SNP caller on the inference of putative variants. Due to sugarcane's aneuploidy at the locus level, it was not possible to define an exclusive ploidy to be used for the entire dataset. Due to this difficulty, we tested all the possible ploidy configurations with GATK and FreeBayes to check the influence of this parameter on the final set. The quantities of markers were similar (Supplementary Table S10). Furthermore, these positions were the same for different ploidies, with only the quantity of detected variants and related allelic dosages changing.

Supplementary Table S10. Identification of SNPs considering different ploidies and using the methyl-filtered (MF) genome of sugarcane cultivar SP70-1143, GATK, FreeBayes and SAMtools.

	GATK	FreeBayes	SAMtools
Ploidy 2	61,604	77,079	353,715
Ploidy 4	61,481	77,079	-
Ploidy 6	61,441	77,079	-
Ploidy 8	61,317	77,079	-

Ploidy 10	62,083	76,932	-
Ploidy 12	62,059	76,932	-
Ploidy 14	0	76,560	-
Ploidy 16	0	76,008	-
Ploidy 18	0	73,110	-
Ploidy 20	0	72,391	-

Although there was only a small discrepancy among the positions of the analyzed variants among ploidy configurations, the quantities of missing data were very different (Supplementary Table S11). As the defined ploidy level increased, the quantity of missing data also increased. This fact may be explained by the depth required by these tools to characterize a haplotype. To increase the reliability of the dataset, the intersection of these positions between different ploidy configurations (2-12) was examined, and individual genotypes were further evaluated.

Supplementary Table S11. Different missing data (NA) distributions across the used ploidy range using the methyl-filtered (MF) genome of sugarcane cultivar SP70-1143, GATK, FreeBayes and SAMtools.

Ploidy	Tool	SNPs	Maximum of 90% NAs	Maximum of 75% NAs	Maximum of 50% NAs	Maximum of 25% NAs
2	GATK	61,604	40,486	35,947	27,504	16,578
	FreeBayes	77,079	73,668	69,650	60,335	49,492
	SAMtools	353,715	227,844	195,180	156,848	123,678
4	GATK	61,481	40,441	35,901	27,463	16,548
	FreeBayes	77,079	73,668	69,650	60,335	49,492
6	GATK	61,441	40,413	35,865	27,427	16,531
	FreeBayes	77,079	73,668	69,650	60,335	49,492
8	GATK	61,317	40,347	35,803	27,373	16,491
	FreeBayes	77,079	73,668	69,650	60,335	49,492
10	GATK	62,083	25,861	16,251	11,190	7,543
	FreeBayes	76,932	73,527	69,526	60,236	49,412
12	GATK	62,059	25,882	16,260	11,200	7,546
	FreeBayes	76,932	73,527	69,526	60,236	49,412

7. Cross-Validation Results with Allele Dosages

The selection of markers based on the defined criteria was organized into allele dosages (ADs) and allele proportions (APs) with the following subsets:

1. All (total SNP dataset);
2. FS1 (feature selection 1 - gradient tree boosting);
3. FS2 (feature selection 2 - L1-based SVC);
4. FS3 (feature selection 3 - extra trees);
5. FS4 (feature selection 4 - F statistic from ANOVA); and
6. FS5 (feature selection 5 - random forests).

Accuracy, recall, precision and specificity metrics for each subset of markers are provided in Supplementary Tables S12 to S17. The first comparison that we performed was to evaluate the appropriateness of using the ADs or APs for this task. For each measure, we calculated the percentage increase caused by using APs instead of ADs, coloring these percentages in blue (increase), red (decrease) or black (maintenance).

Supplementary Table S12. Cross-validation results when using the entire dataset of SNPs organized as allele dosages (AD) and allele proportions (AP). The percentages of gain or loss when using APs instead of ADs are colored in blue and red, respectively. The tested machine learning (ML) models were adaptive boosting (AB), decision tree (DT), Gaussian process (GP), K-nearest neighbor (KNN), multilayer perceptron (MLP), Gaussian naive Bayes (GNB), random forest (RF), and support vector machine (SVM).

Performances								
Model	Accuracy (AD)	Accuracy (AP)	Recall (AD)	Recall (AP)	Precision (AD)	Precision (AP)	Specificity (AD)	Specificity (AP)
AB	52.72	58.75 **(+11%)**	53.69	60.14 **(+12%)**	70.74	75.12 **(+6%)**	50.55	55.66 **(+10%)**
DT	51.95	55.08 **(+6%)**	53.18	55.32 **(+4%)**	69.99	73.04 **(+4%)**	49.23	54.53 **(+11%)**
GP	31.27	68.94 **(+20%)**	0.41	99.80 **(+24241%)**	100.00	69.01 **(-31%)**	100.00	0.23 **(-100%)**
KNN	68.97	68.60 **(-1%)**	99.95	98.97 **(-1%)**	68.99	69.00 **(+0%)**	0.00	1.00 **(+0%)**
MLP	54.58	49.26 **(-10%)**	59.69	48.14 **(-19%)**	70.05	68.96 **(-2%)**	43.19	51.75 **(+20%)**
GNB	50.33	47.85 **(-5%)**	42.36	39.47 **(-7%)**	74.70	72.40 **(-3%)**	68.06	66.49 **(-2%)**
RF	48.55	50.12 **(+3%)**	41.64	43.70 **(+4%)**	71.99	73.21 **(+2%)**	63.92	64.40 **(+0%)**
SVM	47.61	53.91 **(+13%)**	35.99	52.87 **(+47%)**	75.13	72.89 **(-3%)**	73.47	56.21 **(-23%)**
Mean	51.14	54.50	47.77	54.10	71.37	72.65	57.24	55.10

		(+7%)		(+13%)		(+2%)		(-4%)

Supplementary Table S13. Cross-validation results when using the dataset of SNPs obtained with gradient tree boosting (FS1) and organized as allele dosages (AD) and allele proportions (AP). The percentages of gain or loss when using APs instead of ADs are colored in blue and red, respectively. The tested machine learning (ML) models were adaptive boosting (AB), decision tree (DT), Gaussian process (GP), K-nearest neighbor (KNN), multilayer perceptron (MLP), Gaussian naive Bayes (GNB), random forest (RF), and support vector machine (SVM).

**Performances**								
Model	Accuracy (AD)	Accuracy (AP)	Recall (AD)	Recall (AP)	Precision (AD)	Precision (AP)	Specificity (AD)	Specificity (AP)
AB	71.49	77.50 **(+8%)**	72.36	78.07 **(+8%)**	84.09	87.97 **(+4%)**	69.53	76.23 **(+10%)**
DT	61.20	66.23 **(+8%)**	61.56	67.12 **(+9%)**	77.58	80.70 **(+4%)**	60.40	64.26 **(+6%)**
GP	69.83	81.11 **(+16%)**	98.49	88.63 **(-10%)**	70.00	84.71 **(+21%)**	6.02	64.38 **(+969%)**
KNN	69.24	70.27 **(+1%)**	99.78	98.00 **(-2%)**	69.23	70.46 **(+2%)**	1.25	8.53 **(+582%)**
MLP	83.13	78.26 **(-6%)**	86.13	80.49 **(-7%)**	89.07	87.03 **(-2%)**	76.47	73.30 **(-4%)**
GNB	83.83	86.37 **(+3%)**	79.87	83.60 **(+5%)**	96.03	96.15 **(+0%)**	92.64	92.55 **(+0%)**
RF	63.29	68.60 **(+8%)**	57.38	63.42 **(+11%)**	84.43	87.66 **(+4%)**	76.43	80.13 **(+5%)**
SVM	70.56	78.11 **(+10%)**	61.19	80.59 **(+32%)**	94.08	86.75 **(-8%)**	91.43	72.58 **(-21%)**
Mean	70.20	77.80 **(+11%)**	76.12	80.54 **(+6%)**	84.26	86.89 **(+3%)**	72.98	72.94 **(+0%)**

Supplementary Table S14. Cross-validation results when using the dataset of SNPs obtained with the L1-based support vector classification system (FS2) and organized as allele dosages (AD) and allele proportions (AP). The percentages of gain or loss when using APs instead of ADs are colored in blue and red, respectively. The tested machine learning (ML) models were adaptive boosting (AB), decision tree (DT), Gaussian process (GP), K-nearest neighbor (KNN), multilayer perceptron (MLP), Gaussian naive Bayes (GNB), random forest (RF), and support vector machine (SVM).

Performances								
Model	Accuracy (AD)	Accuracy (AP)	Recall (AD)	Recall (AP)	Precision (AD)	Precision (AP)	Specificity (AD)	Specificity (AP)
AB	70.85	75.98 **(+7%)**	71.07	76.59 **(+8%)**	84.22	87.04 **(+3%)**	70.36	74.60 **(+6%)**
DT	57.18	60.69 **(+6%)**	57.45	61.05 **(+6%)**	74.66	77.21 **(+3%)**	56.58	59.89 **(+6%)**
GP	69.53	88.03 **(+27%)**	96.61	99.80 **(+3%)**	70.32	85.34 **(+21%)**	9.23	61.83 **(+570%)**
KNN	71.29	71.11 **(+0%)**	98.79	99.22 **(+0%)**	70.98	70.72 **(+0%)**	10.06	8.53 **(-20%)**
MLP	90.42	97.71 **(+8%)**	91.56	98.55 **(+8%)**	94.39	98.14 **(+4%)**	87.89	95.85 **(+9%)**
GNB	86.26	88.53 **(+3%)**	86.79	87.15 **(+0%)**	92.83	95.84 **(+3%)**	85.08	91.58 **(+8%)**
RF	60.75	66.02 **(+9%)**	54.12	60.29 **(+11%)**	83.12	86.35 **(+4%)**	75.53	78.77 **(+4%)**
SVM	49.95	92.50 **(+85%)**	29.95	92.05 **(+207%)**	92.37	96.93 **(+5%)**	94.49	93.50 **(-1%)**
Mean	70.19	82.01 **(+17%)**	78.93	89.60 **(+14%)**	83.67	86.70 **(+4%)**	72.95	76.69 **(+5%)**

Supplementary Table S15. Cross-validation results when using the dataset of SNPs obtained with extra trees (FS3) and organized as allele dosages (AD) and allele proportions (AP). The percentages of gain or loss when using APs instead of ADs are colored in blue and red, respectively. The tested machine learning (ML) models were adaptive boosting (AB), decision tree (DT), Gaussian process (GP), K-nearest neighbor (KNN), multilayer perceptron (MLP), Gaussian naive Bayes (GNB), random forest (RF), and support vector machine (SVM).

Performances								
Model	Accuracy (AD)	Accuracy (AP)	Recall (AD)	Recall (AP)	Precision (AD)	Precision (AP)	Specificity (AD)	Specificity (AP)
AB	58.32	62.71 **(+8%)**	58.97	65.08 **(+10%)**	75.27	77.29 **(+3%)**	56.87	57.43 **(+1%)**
DT	53.65	56.06 **(+4%)**	54.29	57.12 **(+5%)**	71.68	73.31 **(+2%)**	52.25	53.70 **(+3%)**
GP	36.86	69.01 **(+87%)**	8.97	100.00 **(+1015%)**	95.06	69.01 **(-27%)**	98.96	0.00 **(-100%)**
KNN	68.96	69.01 **(+0%)**	99.91	99.77 **(+0%)**	69.00	69.07 **(+0%)**	0.06	0.53 **(+783%)**
MLP	73.01	52.99	82.44	52.31	79.28	71.90	52.02	54.49

		**(-27%)**		**(-37%)**		**(-9%)**		**(+5%)**
GNB	67.16	65.51 **(-2%)**	60.49	58.06 **(-4%)**	88.21	87.83 **(+0%)**	82.00	82.09 **(+0%)**
RF	52.53	55.36 **(+5%)**	45.70	48.81 **(+7%)**	75.93	78.34 **(+3%)**	67.74	69.94 **(+3%)**
SVM	61.05	68.27 **(+12%)**	51.76	68.08 **(+32%)**	86.32	82.88 **(-4%)**	81.74	68.70 **(-16%)**
Mean	59.69	64.11 **(+7%)**	56.63	61.57 **(+9%)**	77.61	75.3 **(-3%)**	62.31	55.96 **(-10%)**

Supplementary Table S16. Cross-validation results when using the dataset of SNPs obtained with the F statistic from ANOVA (FS4) and organized as allele dosages (AD) and allele proportions (AP). The percentages of gain or loss when using APs instead of ADs are colored in blue and red, respectively. The tested machine learning (ML) models were adaptive boosting (AB), decision tree (DT), Gaussian process (GP), K-nearest neighbor (KNN), multilayer perceptron (MLP), Gaussian naive Bayes (GNB), random forest (RF), and support vector machine (SVM).

**Performances**								
Model	Accuracy (AD)	Accuracy (AP)	Recall (AD)	Recall (AP)	Precision (AD)	Precision (AP)	Specificity (AD)	Specificity (AP)
AB	72.37	73.57 **(+2%)**	72.90	74.86 **(+3%)**	84.92	85.05 **(+0%)**	71.19	70.70 **(-1%)**
DT	58.54	60.27 **(+3%)**	58.69	60.85 **(+4%)**	75.77	76.77 **(+1%)**	58.21	59.00 **(+1%)**
GP	69.68	81.64 **(+17%)**	97.53	99.04 **(+2%)**	70.16	79.43 **(+13%)**	7.66	42.91 **(+460%)**
KNN	72.18	72.53 **(+0%)**	99.25	99.46 **(+0%)**	71.50	71.70 **(+0%)**	11.91	12.58 **(+6%)**
MLP	95.35	86.81 **(-9%)**	96.13	87.91 **(-9%)**	97.10	92.60 **(-5%)**	93.60	84.36 **(-10%)**
GNB	92.89	90.77 **(-2%)**	94.60	90.50 **(-4%)**	95.07	95.89 **(+1%)**	89.08	91.36 **(+3%)**
RF	66.88	66.67 **(0%)**	61.14	61.22 **(+0%)**	87.01	86.54 **(-1%)**	79.68	78.79 **(-1%)**
SVM	94.42	86.61 **(-8%)**	94.59	85.43 **(-10%)**	97.24	94.65 **(-3%)**	94.02	89.25 **(-5%)**
Mean	72.28	77.61 **(+7%)**	94.60	86.67 **(-9%)**	85.97	85.80 **(+0%)**	75.44	74.75 **(-1%)**

Supplementary Table S17. Cross-validation results when using the dataset of SNPs obtained with random forests (FS5) and organized as allele dosages (AD) and allele proportions (AP). The percentages of gain or loss when using APs

instead of ADs are colored in blue and red, respectively. The tested machine learning (ML) models were adaptive boosting (AB), decision tree (DT), Gaussian process (GP), K-nearest neighbor (KNN), multilayer perceptron (MLP), Gaussian naive Bayes (GNB), random forest (RF), and support vector machine (SVM).

**Performances**								
Model	Accuracy (AD)	Accuracy (AP)	Recall (AD)	Recall (AP)	Precision (AD)	Precision (AP)	Specificity (AD)	Specificity (AP)
AB	59.19	66.25 **(+12%)**	59.77	68.16 **(+14%)**	75.97	79.97 **(+5%)**	57.91	62.00 **(+7%)**
DT	54.08	57.40 **(+6%)**	54.90	58.07 **(+6%)**	71.91	74.58 **(+4%)**	52.25	55.92 **(+7%)**
GP	37.25	69.02 **(+85%)**	9.85	100.00 **(+915%)**	92.66	69.02 **(-26%)**	98.26	0.057 **(-100%)**
KNN	68.99	69.12 **(+0%)**	99.98	99.94 **(+0%)**	69.00	69.10 **(+0%)**	0.00	0.49 **(+0%)**
MLP	72.43	63.11 **(-13%)**	82.87	65.76 **(-21%)**	78.41	77.38 **(-1%)**	49.19	57.21 **(+16%)**
GNB	65.78	69.22 **(+5%)**	59.21	61.50 **(+4%)**	87.07	90.96 **(+4%)**	80.42	86.40 **(+7%)**
RF	52.35	57.90 **(+11%)**	45.65	51.79 **(+13%)**	75.63	80.19 **(+6%)**	67.25	71.51 **(+6%)**
SVM	51.93	68.22 **(+31%)**	36.66	67.75 **(+85%)**	85.29	83.06 **(-3%)**	85.92	69.25 **(-19%)**
Mean	56.64	67.24 **(+19%)**	57.06	66.76 **(+17%)**	77.19	78.68 **(+2%)**	62.58	59.61 **(-5%)**

Pairwise comparisons (ADs versus APs) between the metrics of each model for the different configurations are provided in Supplementary Table S18. The performance of APs was superior to that of ADs for all the metrics.

Supplementary Table S18. Comparison of machine learning (ML) predictive performance between datasets obtained with allele proportions (APs) and allele dosages (ADs).

Performance	Accuracy	Recall	Precision	Specificity
AP > AD	33 (69%)	30 (63%)	25 (52%)	28 (58%)
AD > AP	10 (21%)	12 (25%)	15 (31%)	15 (31%)
AD = AP	5 (10%)	6 (12%)	8 (17%)	5 (11%)

Another important step was to establish which feature selection (FS) techniques were appropriate for correctly predicting the different phenotypes. The superior boundaries for each confidence interval created for the mean values are

shown in Supplementary Table S19. Accuracy was the only measure that did not have a significant p-value for the Shapiro-Wilk normality test (0.09678); thus, to quantify the confidence interval, we considered a normal distribution. For recall, precision and specificity, on the other hand, we used a Wilcoxon test to calculate these boundaries.

Supplementary Table S19. Confidence intervals' superior boundaries calculated for the performance metrics used to evaluate machine learning models when using SNPs as allele proportions.

	Accuracy	Recall	Precision	Specificity
95%	69.72	76.48	82.62	70.36
99%	70.61	77.73	83.25	71.84
99.9%	71.67	79.09	84.06	73.54

Using these values, we counted the results in each FS that exceeded these boundaries to check for significant improvements in performances. These counts are listed in Supplementary Tables S20-S23. Using different restrictions on the confidence intervals, we reached the same conclusions: FS1, FS2 and FS4 outperformed the other methods.

Supplementary Table S20. Model accuracy performance based on the obtained confidence intervals' superior boundaries, considering the SNPs organized as allele proportions. For each feature selection (FS) technique, the quantity of measures that outperformed the calculated superior boundaries are presented below. The FS techniques are organized as FS1 (gradient tree boosting), FS2 (L1-based support vector classification system), FS3 (extra trees), FS4 (F statistic from ANOVA), and FS5 (random forests).

	All	FS1	FS2	FS3	FS4	FS5
95%	0 (0%)	11 (69%)	10 (63%)	1 (6%)	11 (69%)	1 (6%)
99%	0 (0%)	8 (50%)	10 (63%)	1 (6%)	11 (69%)	1 (6%)
99.9%	0 (0%)	7 (44%)	7 (44%)	1 (6%)	11 (69%)	1 (6%)

Supplementary Table S21. Model recall performance based on the obtained confidence intervals' superior boundaries, considering the SNPs organized as allele proportions. For each feature selection (FS) technique, the quantity of measures that outperformed the calculated superior boundaries are presented

below. The FS techniques are organized as FS1 (gradient tree boosting), FS2 (L1-based support vector classification system), FS3 (extra trees), FS4 (F statistic from ANOVA), and FS5 (random forests).

	All	FS1	FS2	FS3	FS4	FS5
95%	3 (19%)	10 (63%)	10 (63%)	4 (25%)	10 (63%)	4 (25%)
99%	3 (19%)	10 (63%)	9 (56%)	4 (25%)	10 (63%)	4 (25%)
99.9%	3 (19%)	9 (56%)	9 (56%)	4 (25%)	10 (63%)	4 (25%)

Supplementary Table S22. Model precision performance based on the obtained confidence intervals' superior boundaries, considering the SNPs organized as allele proportions. For each feature selection (FS) technique, the quantity of measures that outperformed the calculated superior boundaries are presented below. The FS techniques are organized as FS1 (gradient tree boosting), FS2 (L1-based support vector classification system), FS3 (extra trees), FS4 (F statistic from ANOVA), and FS5 (random forests).

	All	FS1	FS2	FS3	FS4	FS5
95%	1 (6%)	11 (69%)	11 (69%)	5 (31%)	10 (63%)	5 (31%)
99%	1 (6%)	11 (69%)	10 (63%)	4 (25%)	10 (63%)	4 (25%)
99.9%	1 (6%)	11 (69%)	10 (63%)	4 (25%)	10 (63%)	4 (25%)

Supplementary Table S23. Model specificity performance based on the obtained confidence intervals' superior boundaries, considering the SNPs organized as allele proportions. For each feature selection (FS) technique, the quantity of measures that outperformed the calculated superior boundaries are presented below. The FS techniques are organized as FS1 (gradient tree boosting), FS2 (L1-based support vector classification system), FS3 (extra trees), FS4 (F statistic from ANOVA), and FS5 (random forests).

	All	FS1	FS2	FS3	FS4	FS5
95%	2 (13%)	9 (56%)	9 (56%)	4 (25%)	10 (63%)	5 (31%)
99%	2 (13%)	9 (56%)	9 (56%)	4 (25%)	8 (50%)	4 (25%)
99.9%	1 (6%)	7 (44%)	9 (56%)	4 (25%)	8 (50%)	4 (25%)

The distribution of the different metrics for each FS can be visualized in Supplementary Figs. S8-S11, together with the groups identified by the Tukey test. The accuracy, recall and precision metrics were significantly different based on ANOVA. Supplementary Fig. S8 shows the group with the highest performance (FS1,

FS2 and FS4). This same behavior is shown in the boxplots in Supplementary Figs. S9 and S10, with the exception that more groups were formed. However, considering the separation identified by the Tukey test of accuracy metrics, the recall and precision groups are divisions of the previous groups with a significant but small difference.

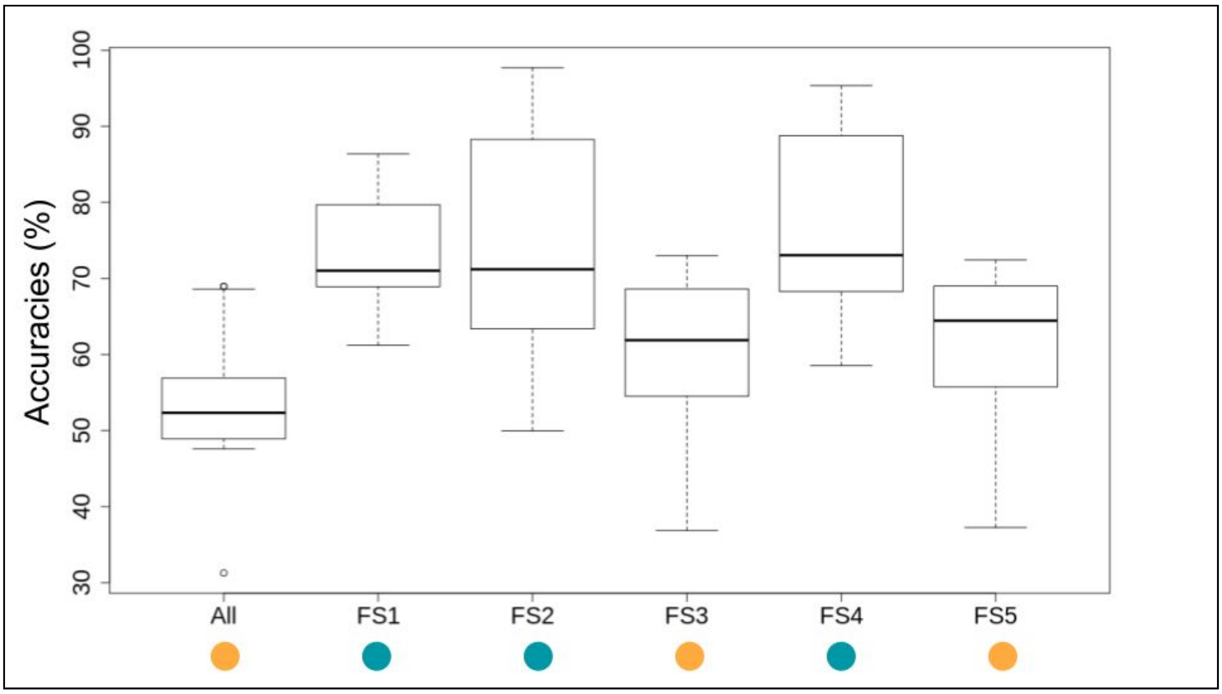

Supplementary Fig. S8. Accuracy distribution for each subset created based on the different feature selection (FS) techniques, considering the SNPs organized as allele proportions. The colors are based on the groups identified with multiple comparisons by Tukey's test (ANOVA p-value of 0.000000000000455) and the FS approaches organized as FS1 (gradient tree boosting), FS2 (L1-based support vector classification system), FS3 (extra trees), FS4 (F statistic from ANOVA), and FS5 (random forests).

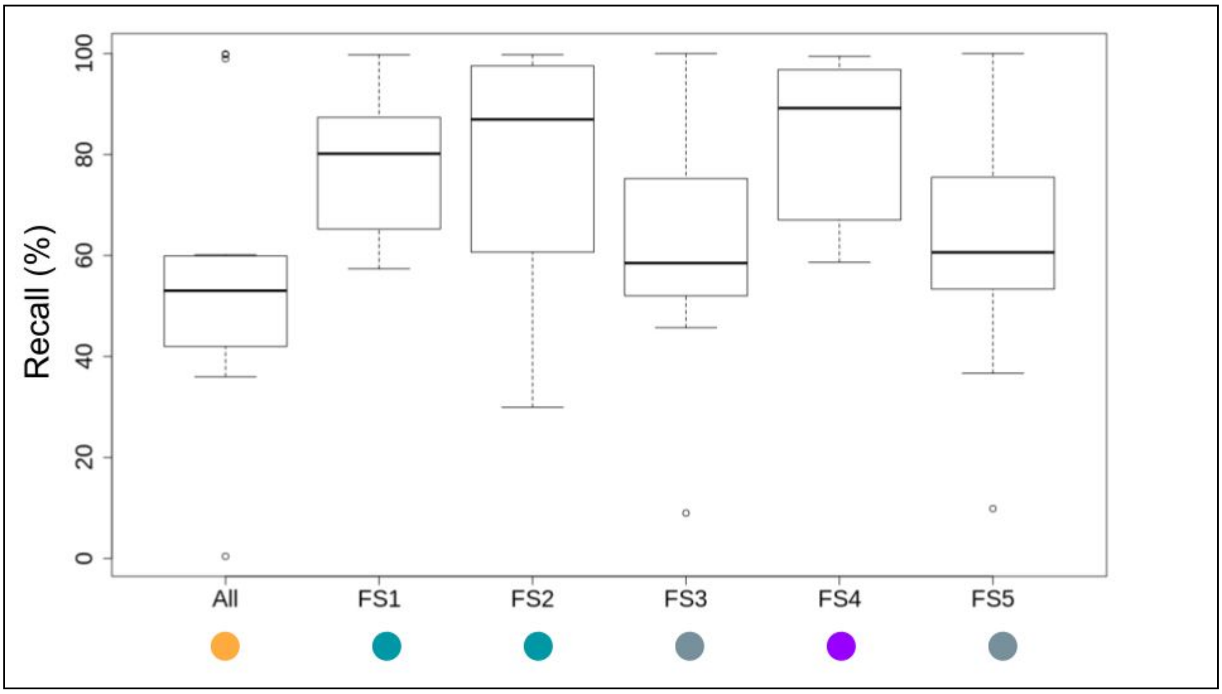

Supplementary Fig. S9. Recall metric distribution for each subset created based on the different feature selection (FS) techniques, considering the SNPs organized as allele proportions. The colors are based on the groups identified with multiple comparisons by Tukey's test (ANOVA p-value of 0.000005375) and the FS approaches organized as FS1 (gradient tree boosting), FS2 (L1-based support vector classification system), FS3 (extra trees), FS4 (F statistic from ANOVA), and FS5 (random forests).

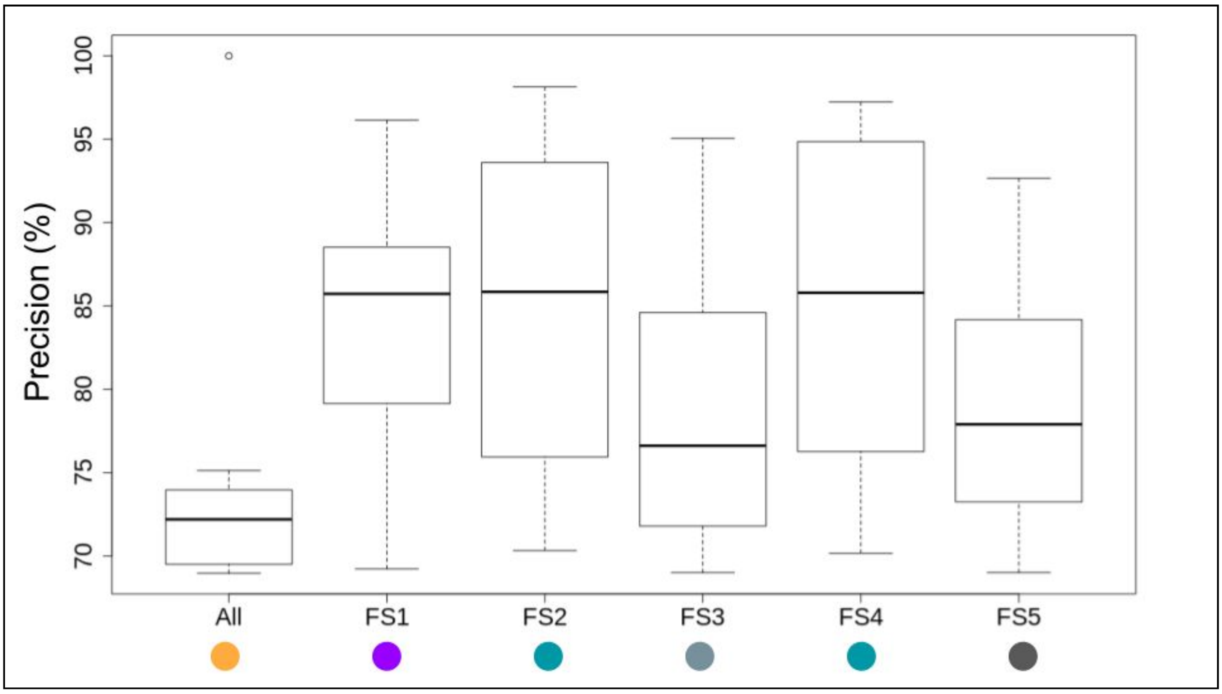

Supplementary Fig. S10. Precision metric distribution for each subset created based on the different feature selection (FS) techniques, considering the SNPs

organized as allele proportions. The colors are based on the groups identified with multiple comparisons by Tukey's test (ANOVA p-value of 0.000000673698) and the FS approaches organized as FS1 (gradient tree boosting), FS2 (L1-based support vector classification system), FS3 (extra trees), FS4 (F statistic from ANOVA), and FS5 (random forests).

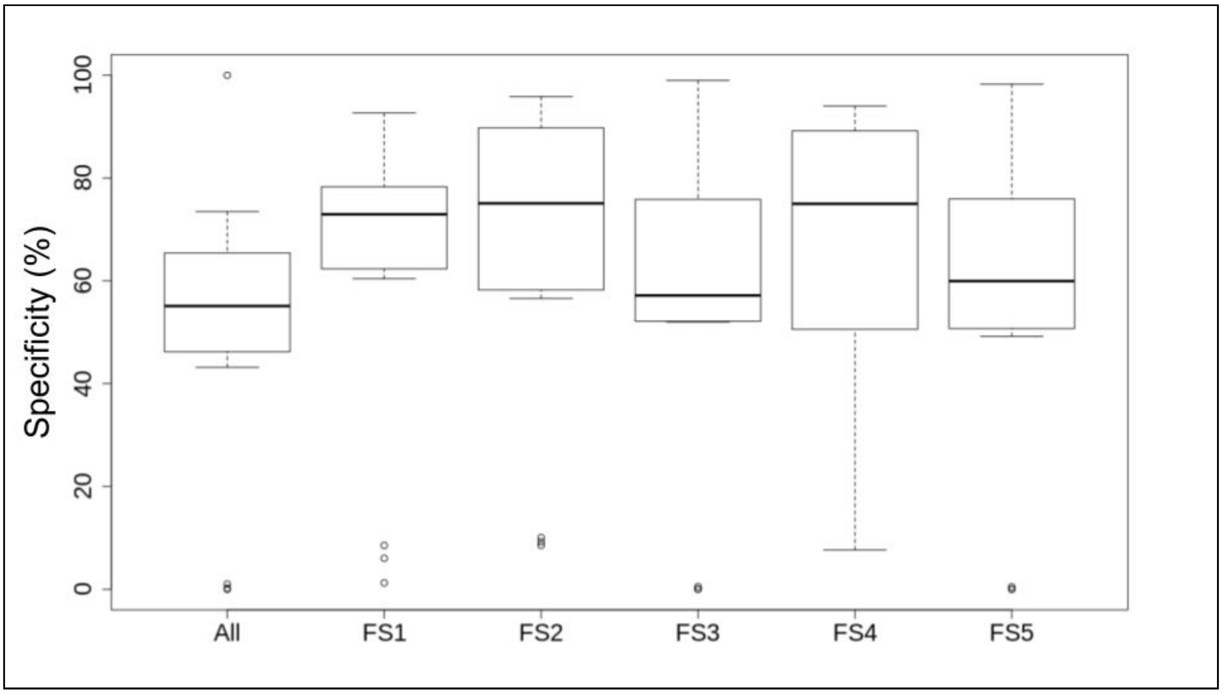

Supplementary Fig. S11. Specificity metric distribution for each subset created based on the different feature selection (FS) techniques (ANOVA p-value of 0.0656), considering the SNPs organized as allele proportions. FS approaches are organized as FS1 (gradient tree boosting), FS2 (L1-based support vector classification system), FS3 (extra trees), FS4 (F statistic from ANOVA), and FS5 (random forests).

Considering FS1, FS2 and FS4 as the most promising strategies for selecting phenotype-associated variants, we selected SNPs identified with all of these methods (6 SNPs) to check for improvements in the evaluated metrics, as shown in Supplementary Table S24. Although individual FS techniques performed better than this dataset, we observed a substantial increase in these metrics when considering the initial dataset with 14,540 SNPs.

Supplementary Table S24. Cross-validation results when using the SNPs as allele proportions selected by all of the best feature selection (FS) techniques, which were gradient tree boosting (FS1), L1-based support vector classification system (FS2), and F statistic from ANOVA (FS4). The mean values were compared with the mean performances of individual FS techniques and the entire set of SNPs (All). The tested machine learning (ML)

models were adaptive boosting (AB), decision tree (DT), Gaussian process (GP), K-nearest neighbor (KNN), multilayer perceptron (MLP), Gaussian naive Bayes (GNB), random forest (RF), and support vector machine (SVM).

**Performances**				
Model	Accuracy	Recall	Precision	Specificity
AB	73.06	72.92	85.90	73.36
DT	69.32	70.14	82.78	67.51
GP	72.57	72.92	85.20	71.79
KNN	70.13	73.55	81.38	62.53
MLP	71.98	72.62	84.60	70.57
GNB	73.32	71.53	87.52	77.28
RF	71.37	68.00	87.75	78.87
SVM	74.20	78.69	83.04	64.21
Mean	72.28	72.77	84.90	71.18
Mean FS1	77.80	80.54	86.89	72.94
Mean FS2	82.01	89.60	86.70	76.69
Mean FS4	77.61	86.67	85.80	74.75
Mean All	54.50	54.10	72.65	55.10

To evaluate the models' performance across the different datasets and check their appropriateness in predicting phenotypic groups, receiver operating characteristic (ROC) curves were constructed, as shown in Supplementary Figs. S12-S19. The model predictive capability changed depending on the dataset configuration, as previously demonstrated.

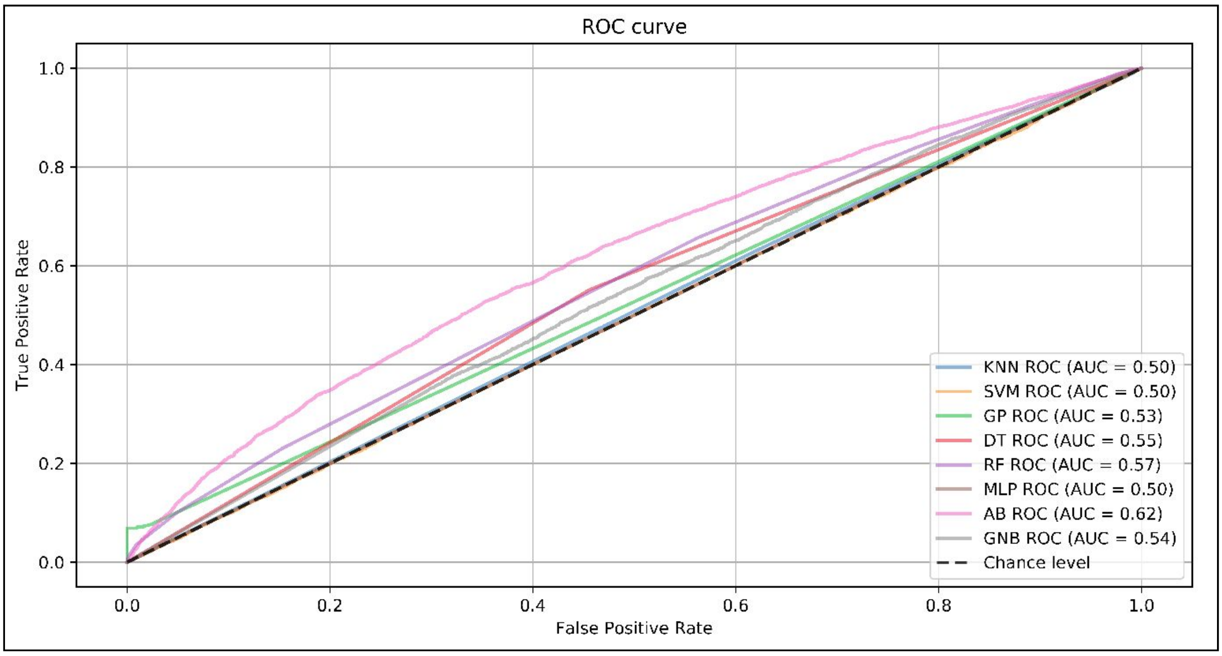

Supplementary Fig. S12. Receiver operating characteristic (ROC) curves of model performance obtained using the entire dataset of SNPs as allele proportions and the tested machine learning (ML) models adaptive boosting (AB), decision tree (DT), Gaussian process (GP), K-nearest neighbor (KNN), multilayer perceptron (MLP), Gaussian naive Bayes (GNB), random forest (RF), and support vector machine (SVM).

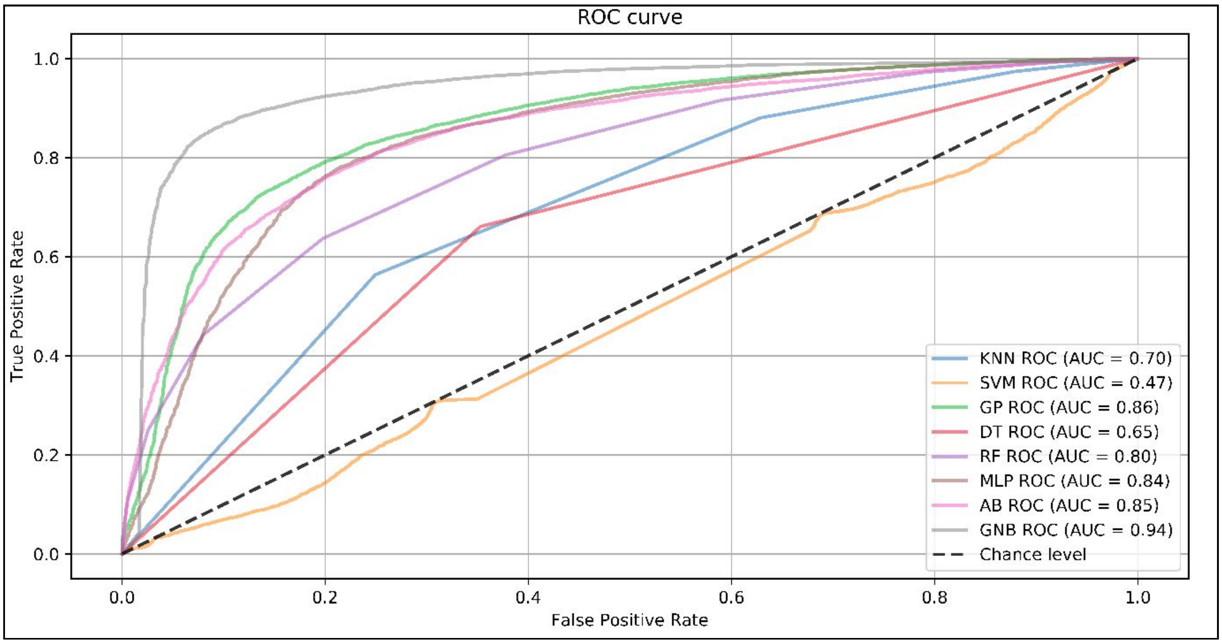

Supplementary Fig. S13. Receiver operating characteristic (ROC) curves of model performance obtained using the dataset of SNPs (as allele proportions) selected by the gradient tree boosting feature selection technique (FS1) and the tested machine learning (ML) models adaptive boosting (AB), decision tree (DT), Gaussian process (GP), K-nearest neighbor (KNN), multilayer perceptron

(MLP), Gaussian naive Bayes (GNB), random forest (RF), and support vector machine (SVM).

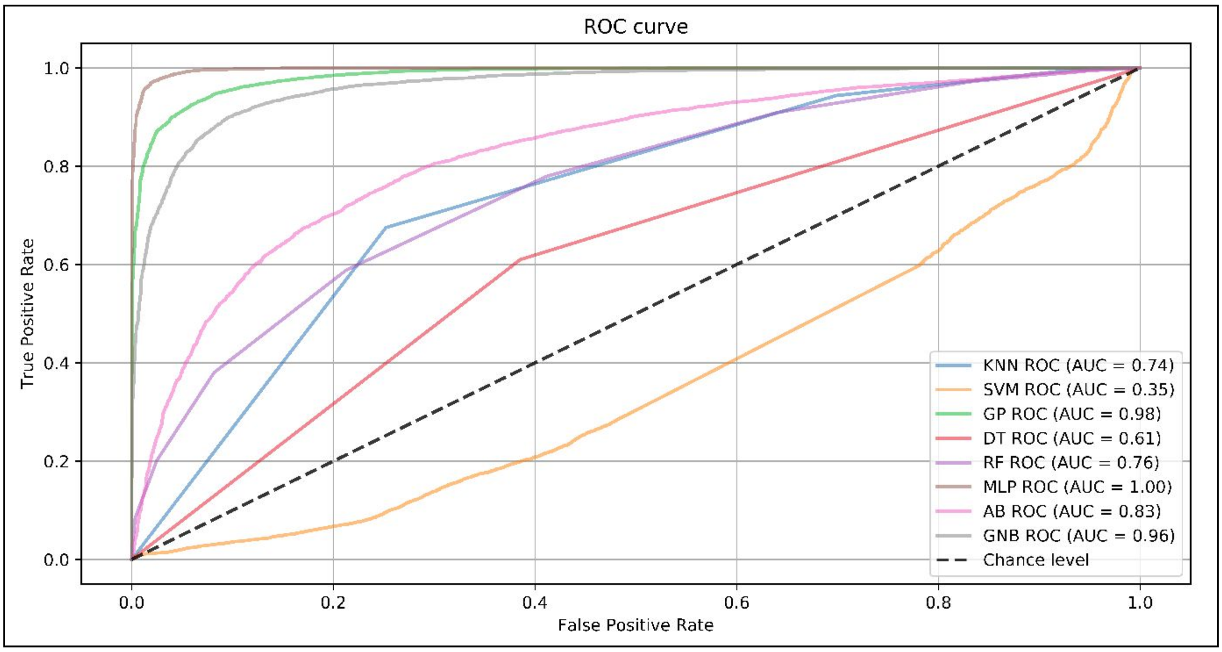

Supplementary Fig. S14. Receiver operating characteristic (ROC) curves of model performance obtained using the dataset of SNPs (as allele proportions) selected by the L1-based support vector classification system feature selection technique (FS2) and the tested machine learning (ML) models adaptive boosting (AB), decision tree (DT), Gaussian process (GP), K-nearest neighbor (KNN), multilayer perceptron (MLP), Gaussian naive Bayes (GNB), random forest (RF), and support vector machine (SVM).

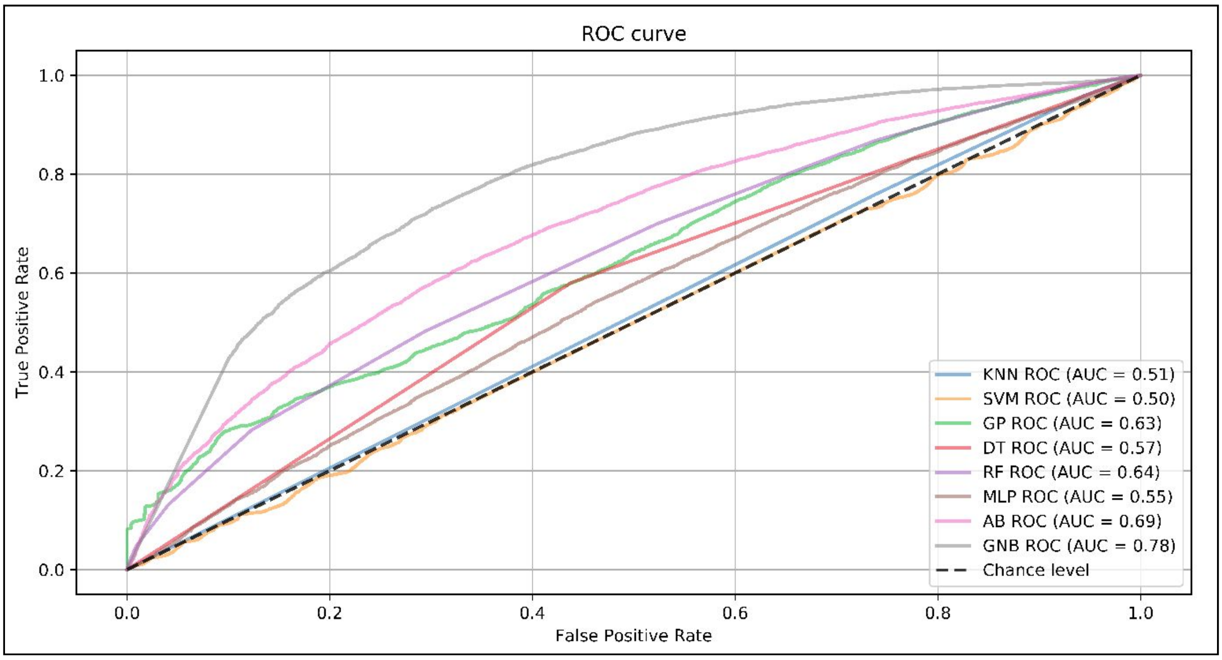

Supplementary Fig. S15. Receiver operating characteristic (ROC) curves of model performance obtained using the dataset of SNPs (as allele proportions) selected by the extra trees feature selection technique (FS3) and the tested machine learning (ML) models adaptive boosting (AB), decision tree (DT), Gaussian process (GP), K-nearest neighbor (KNN), multilayer perceptron (MLP), Gaussian naive Bayes (GNB), random forest (RF), and support vector machine (SVM).

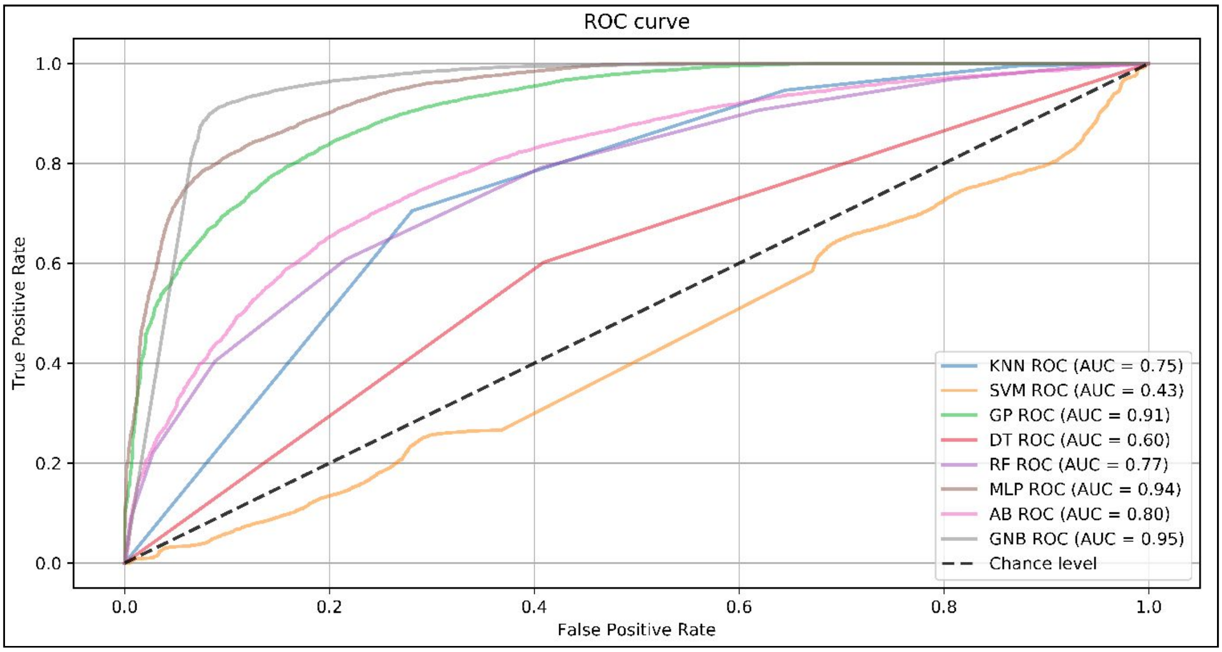

Supplementary Fig. S16. Receiver operating characteristic (ROC) curves of model performance obtained using the dataset of SNPs (as allele proportions) selected by the F statistic from ANOVA feature selection technique (FS4) and the tested machine learning (ML) models adaptive boosting (AB), decision tree

(DT), Gaussian process (GP), K-nearest neighbor (KNN), multilayer perceptron (MLP), Gaussian naive Bayes (GNB), random forest (RF), and support vector machine (SVM).

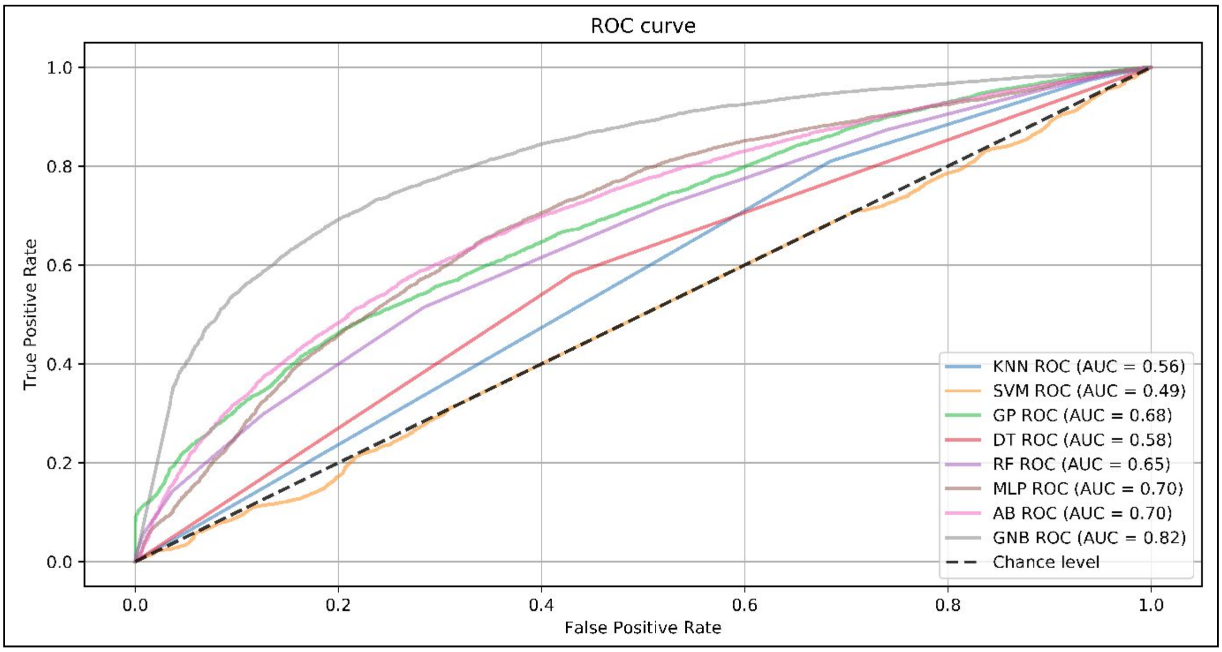

Supplementary Fig. S17. Receiver operating characteristic (ROC) curves of model performance using the dataset of SNPs (as allele proportions) selected by the random forest feature selection technique (FS5) and the tested machine learning (ML) models adaptive boosting (AB), decision tree (DT), Gaussian process (GP), K-nearest neighbor (KNN), multilayer perceptron (MLP), Gaussian naive Bayes (GNB), random forest (RF), and support vector machine (SVM).

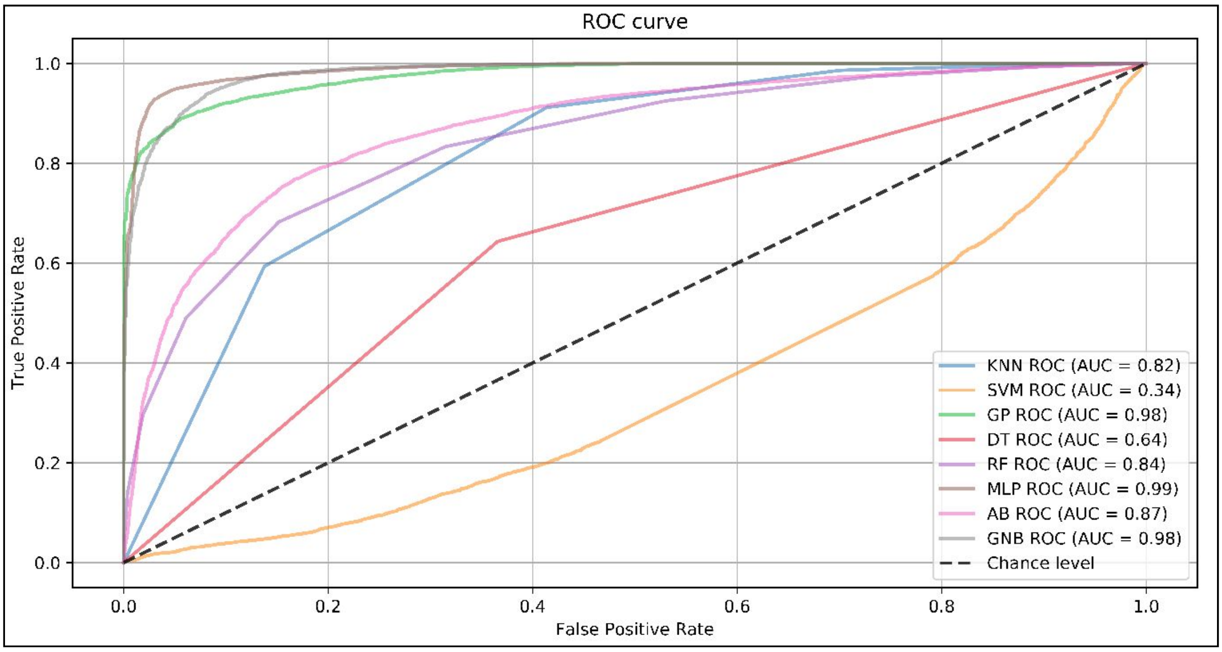

Supplementary Fig. S18. Receiver operating characteristic (ROC) curves of model performance obtained using the dataset of SNPs (as allele proportions) selected by the intersection of at least two of the datasets obtained using the feature selection (FS) techniques: gradient tree boosting (FS1), L1-based support vector classification system (FS2) or the F statistic from ANOVA (FS4). The tested machine learning (ML) models were adaptive boosting (AB), decision tree (DT), Gaussian process (GP), K-nearest neighbor (KNN), multilayer perceptron (MLP), Gaussian naive Bayes (GNB), random forest (RF), and support vector machine (SVM).

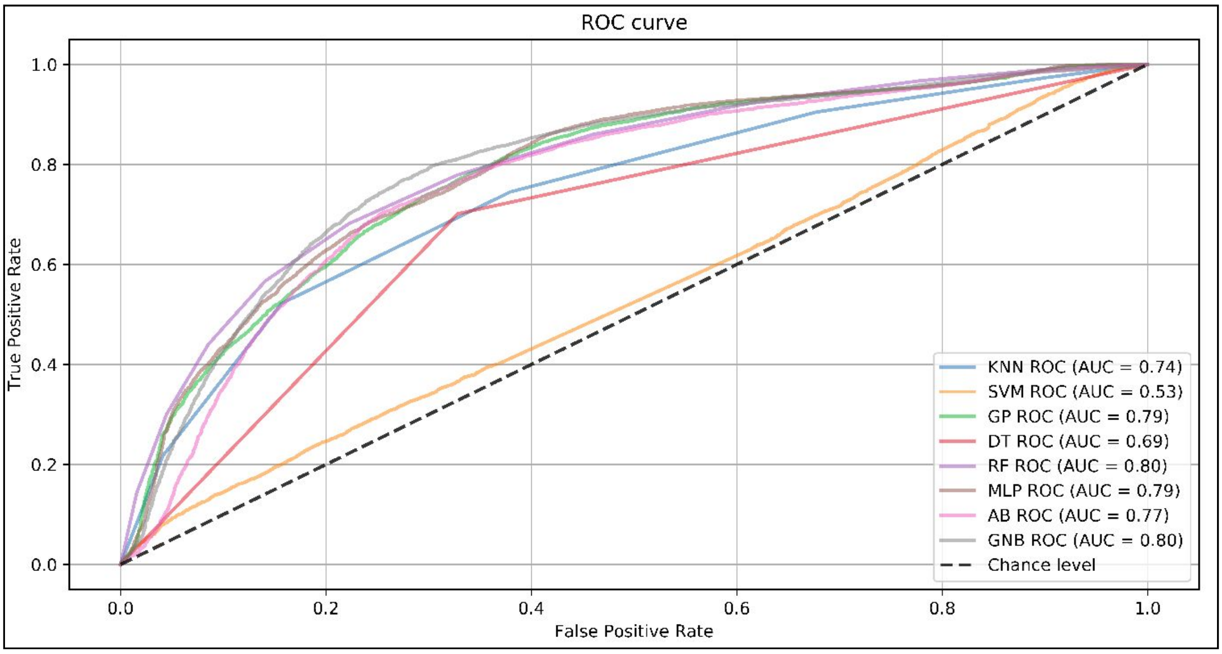

Supplementary Fig. S19. Receiver operating characteristic (ROC) curves of model performance obtained using the dataset of SNPs (as allele proportions)

selected by the intersection of the datasets obtained using the feature selection (FS) techniques: gradient tree boosting (FS1), L1-based support vector classification system (FS2) and the F statistic from ANOVA (FS4). The tested machine learning (ML) models were adaptive boosting (AB), decision tree (DT), Gaussian process (GP), K-nearest neighbor (KNN), multilayer perceptron (MLP), Gaussian naive Bayes (GNB), random forest (RF), and support vector machine (SVM).

According to the ROC curves, the best ML strategies for predicting the phenotypic groups were MLP, GNB and GP. The ROC curves based on these algorithms and the different dataset configurations can be visualized in Supplementary Figs. S20-S22. The best performances were observed for FS2 and the intersection of two FSs (I2).

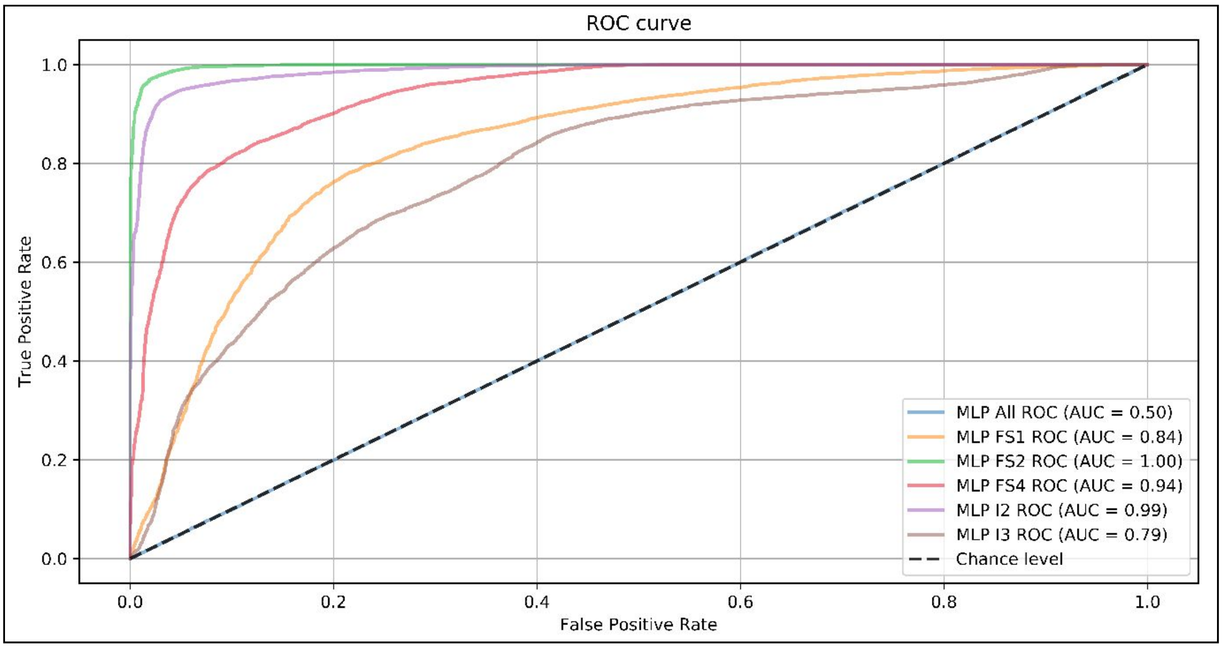

Supplementary Fig. S20. Multilayer perceptron (MLP) results and different dataset configurations (SNPs as allele proportions): All (the entire set of SNPs), FS1 (SNPs selected with gradient tree boosting), FS2 (SNPs selected with the L1-based support vector classification system), FS4 (SNPs selected with the F statistic from ANOVA), I2 (SNPs found with the intersection of at least two methods considering FS1, FS2 and FS4), and I3 (SNPs found with the intersection of FS1, FS2 and FS4).

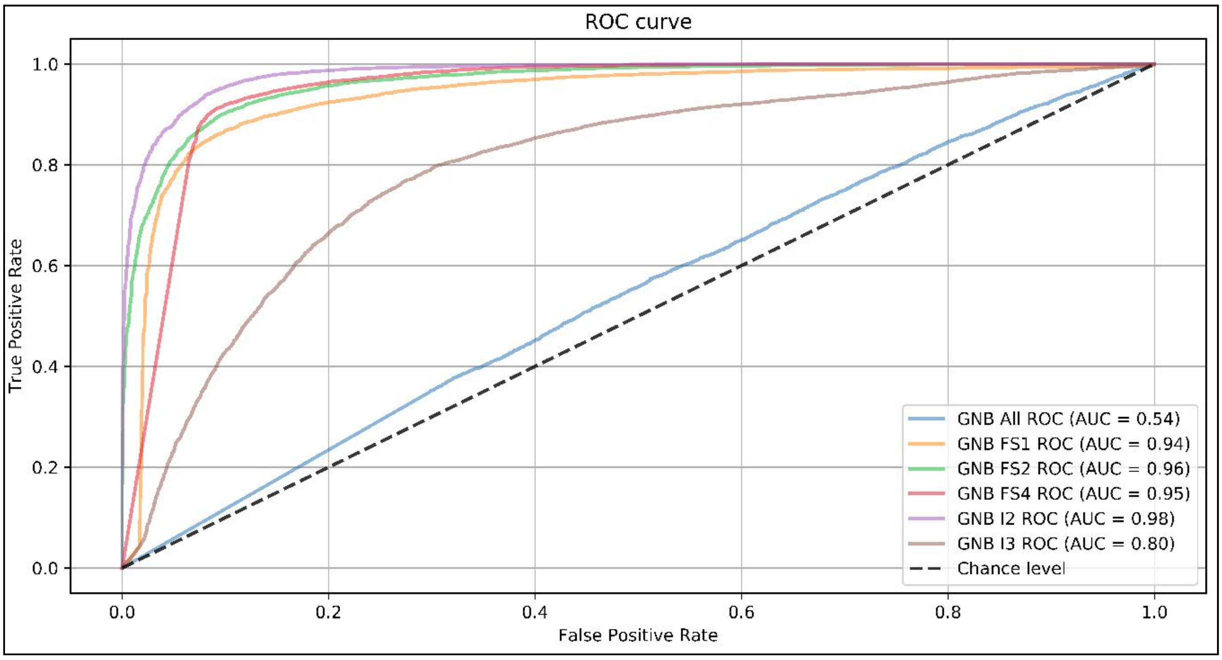

Supplementary Fig. S21. Gaussian naive Bayes (GNB) results and different dataset configurations (SNPs as allele proportions): All (the entire set of SNPs), FS1 (SNPs selected with gradient tree boosting), FS2 (SNPs selected with the L1-based support vector classification system), FS4 (SNPs selected with the F statistic from ANOVA), I2 (SNPs found with the intersection of at least two methods considering FS1, FS2 and FS4), and I3 (SNPs found with the intersection of FS1, FS2 and FS4).

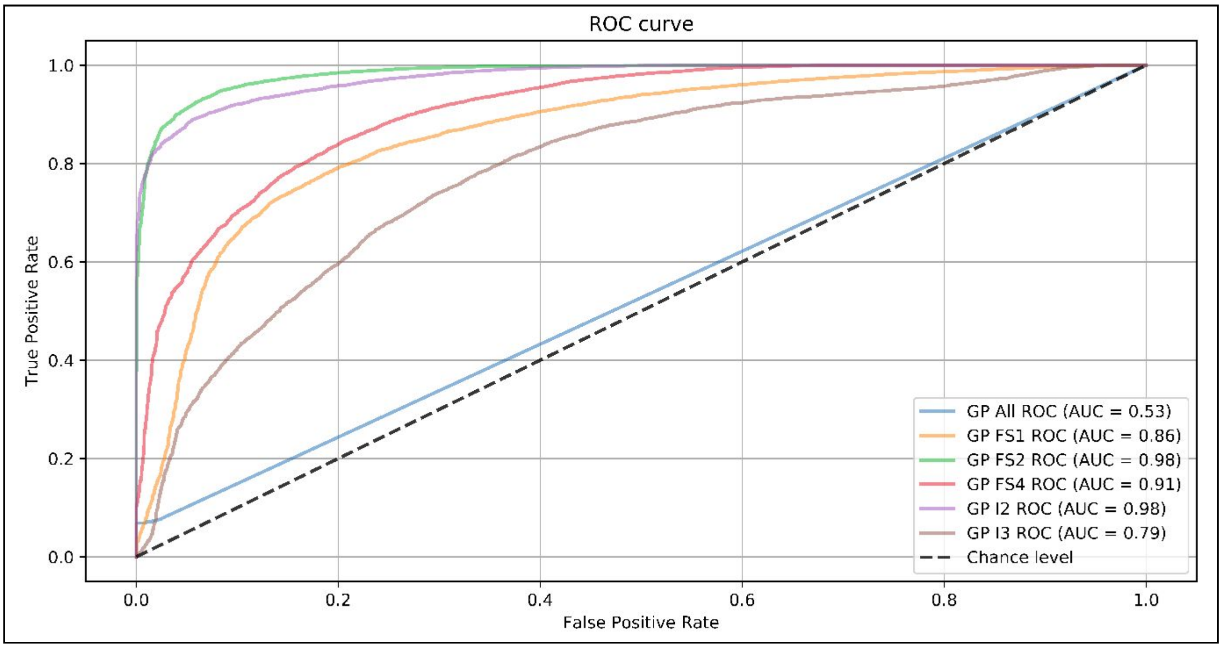

Supplementary Fig. S22. Gaussian process (GP) results and different dataset configurations (SNPs as allele proportions): All (the entire set of SNPs), FS1 (SNPs selected with gradient tree boosting), FS2 (SNPs selected with the L1-based support vector classification system), FS4 (SNPs selected with the F

statistic from ANOVA), I2 (SNPs found with the intersection of at least two methods considering FS1, FS2 and FS4), and I3 (SNPs found with the intersection of FS1, FS2 and FS4).

Using sets of *S. spontaneum* chromosomes with allele specificity, we plotted a physical representation of the organization of subsets of coding DNA sequences (CDSs) related to bacterial artificial chromosomes (BACs) and Inter 2 SNPs.

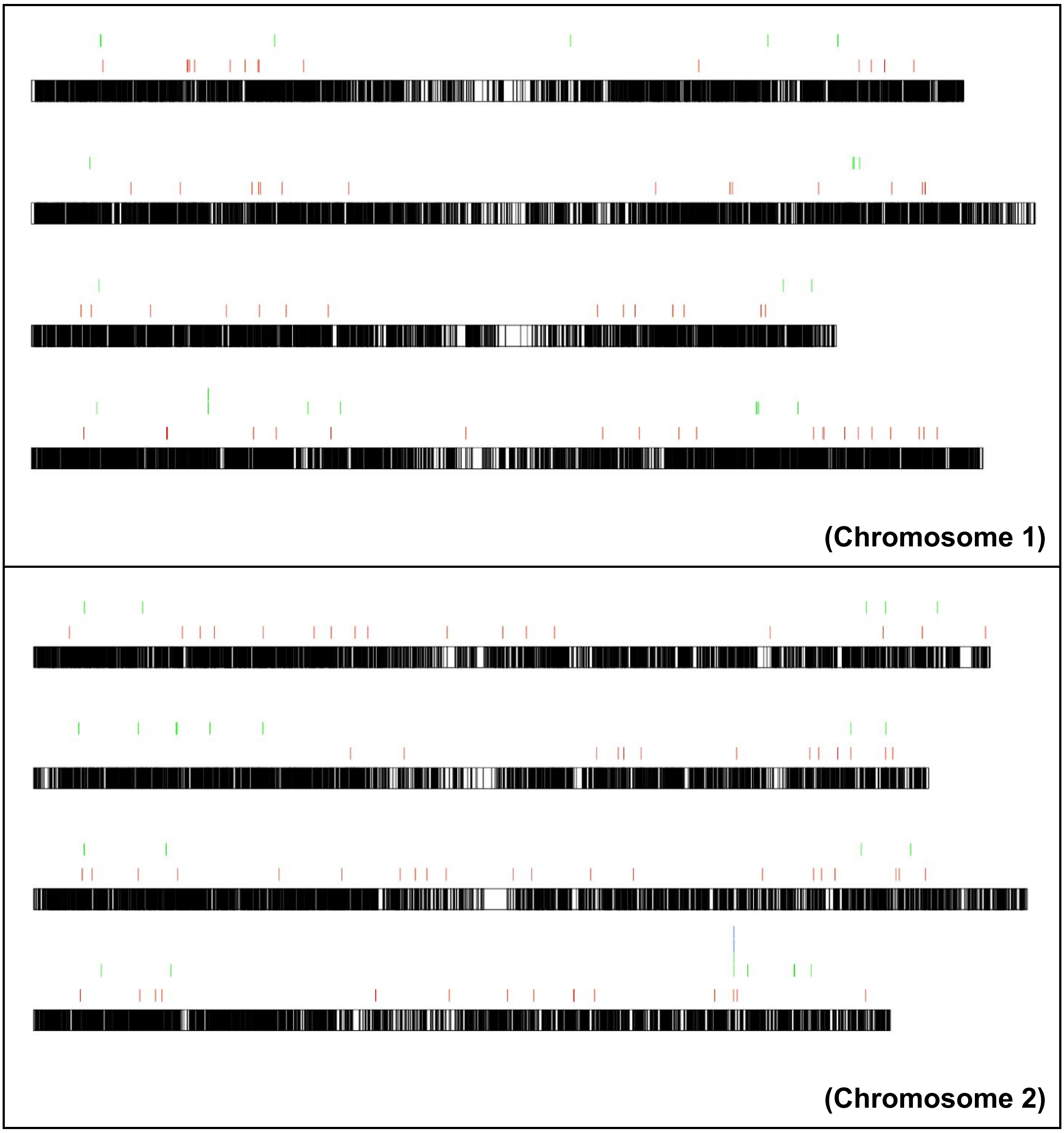

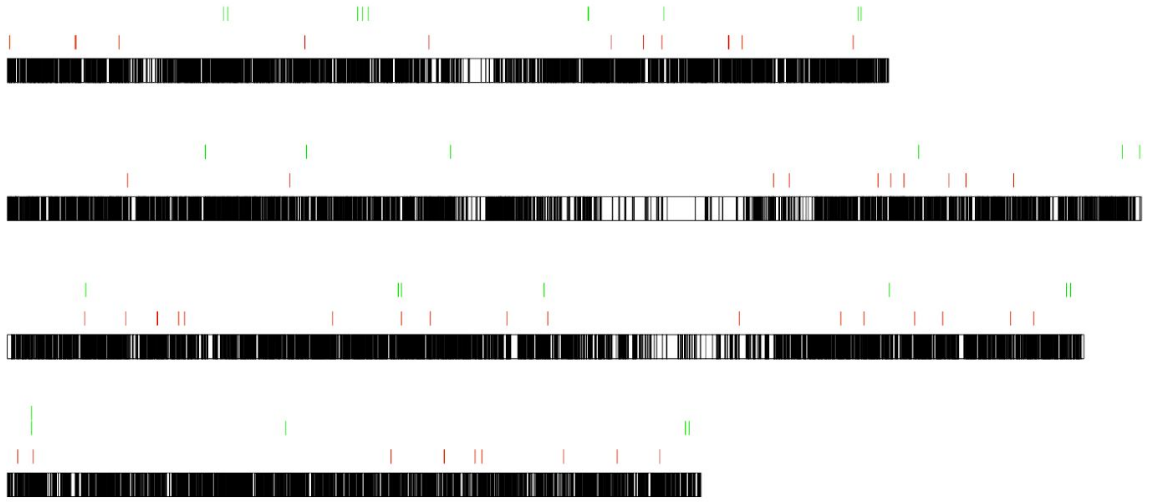

(Chromosome 3)

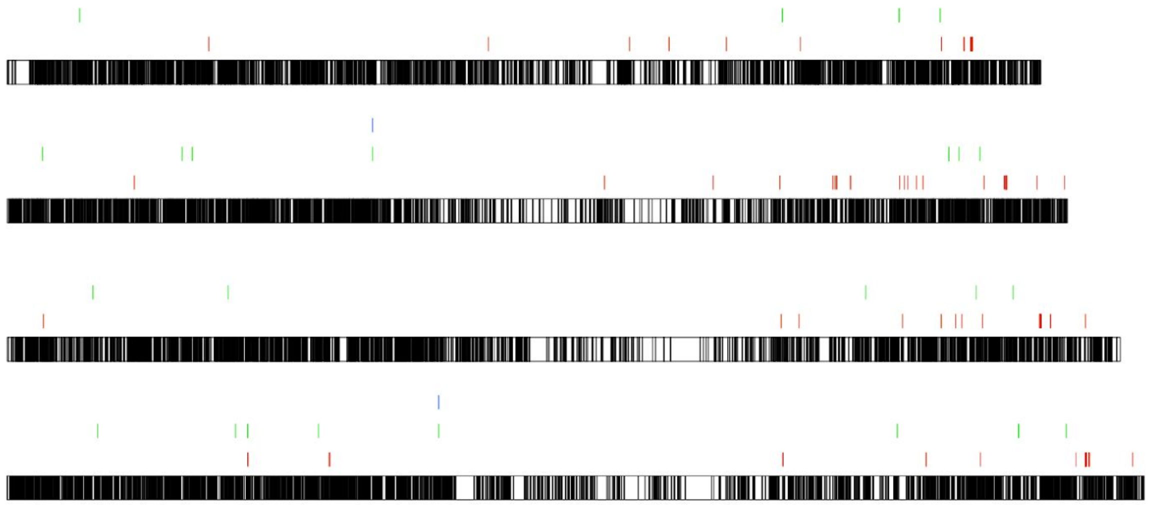

(Chromosome 4)

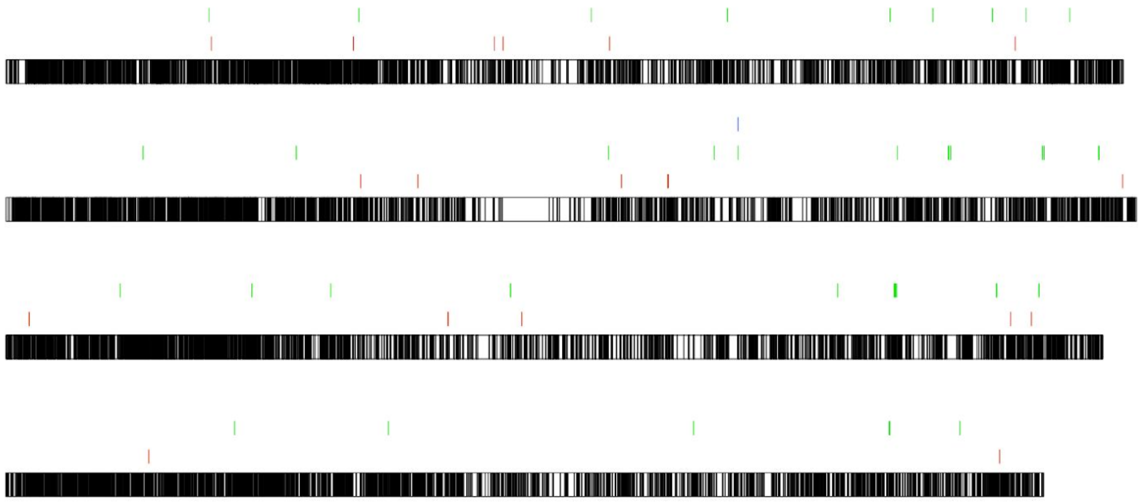

(Chromosome 5)

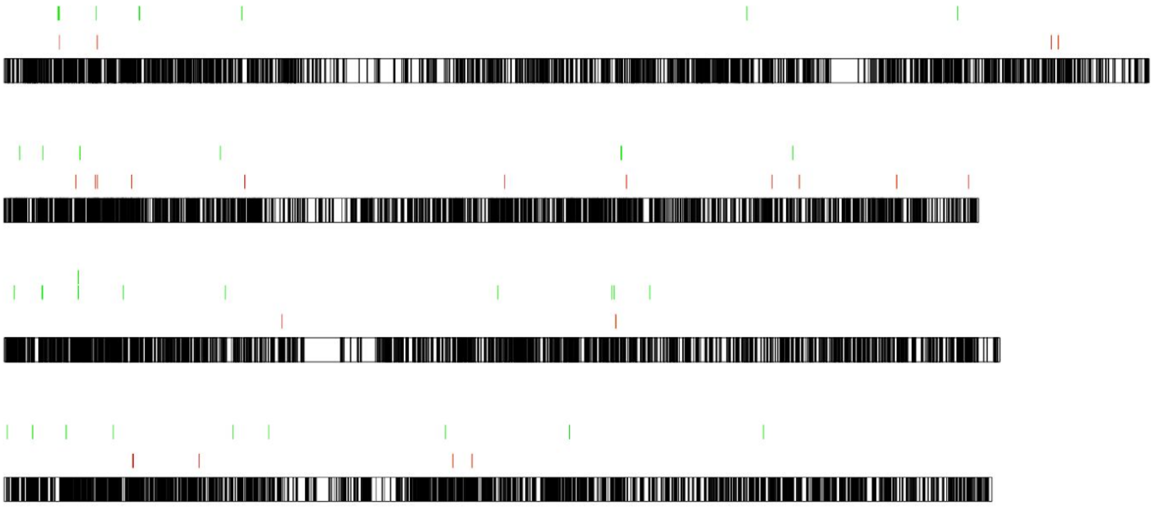

(Chromosome 6)

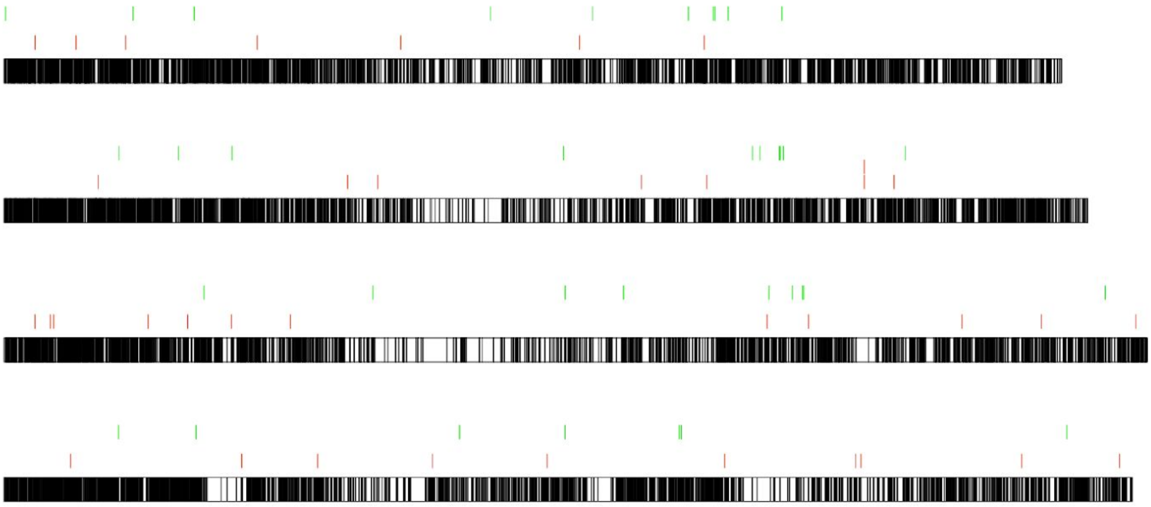

(Chromosome 7)

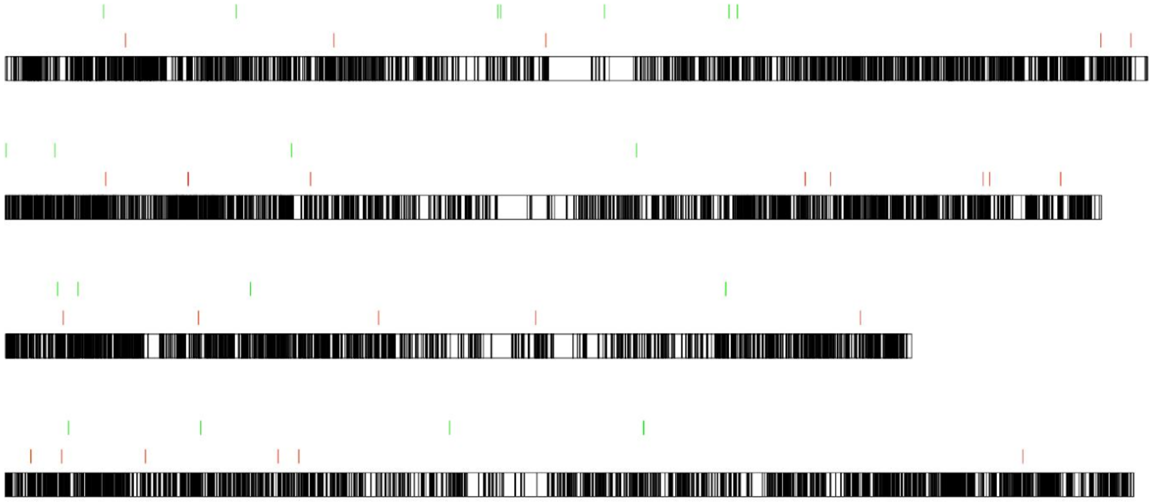

(Chromosome 8)

Supplementary Fig. S23. Representation of coding DNA sequence (CDS) distributions across *S. spontaneum* chromosomes. Each chromosome is in a different block containing the four alleles. This karyotype is colored according to the presence of CDSs (black bands). The locations of CDSs corresponding to brown rust bacterial artificial chromosome (BAC) sequences are colored in red, and the locations of CDSs corresponding to scaffolds containing the SNPs selected by the proposed methodology are colored in green.

There were different *S. spontaneum* CDSs related to (I) BACs and (II) Inter 2 scaffolds, as shown in Supplementary Fig. S23. From these CDSs, it was possible to extract 50 Gene Ontology (GO) categories shared by these two groups and 50 exclusive to (II). We also performed enrichment analysis of these 50 exclusive GOs, the results of which are shown in Supplementary Fig. S24.

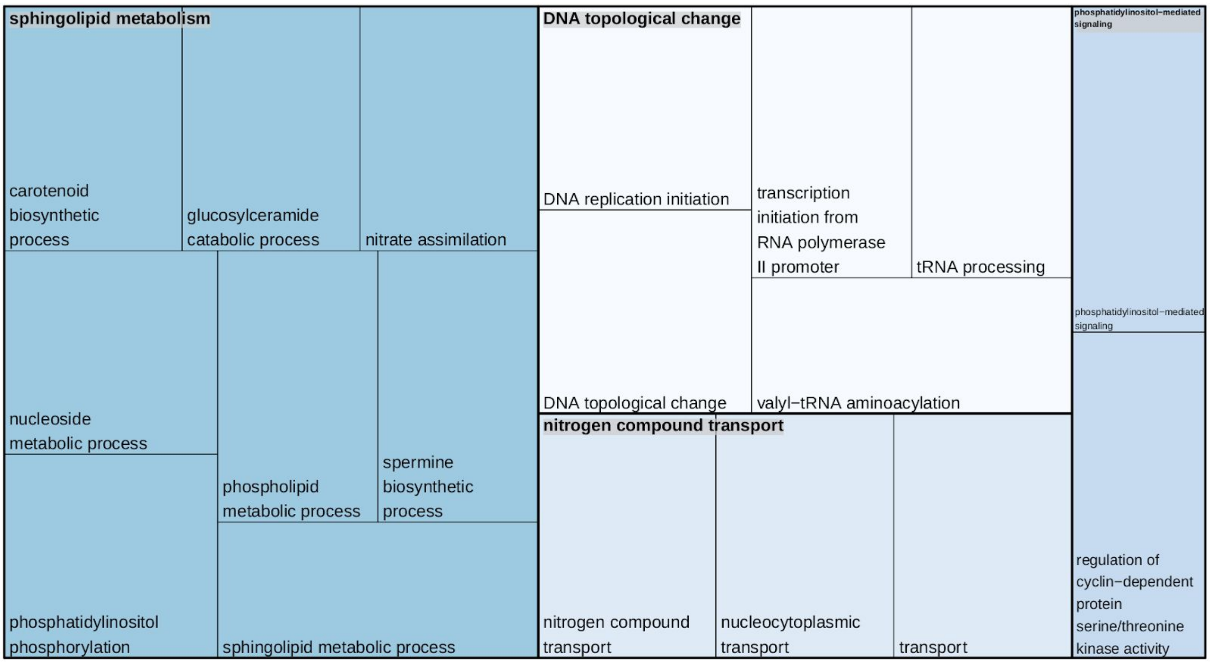

Supplementary Fig. S24. Gene Ontology (GO) categories found among the coding DNA sequences (CDSs) corresponding to scaffolds containing the SNPs selected by the proposed methodology and not in the bacterial artificial chromosomes' (BACs') correspondences.

In addition to GO categories, we also identified corresponding metabolic pathways in (I) and (II), as follows:

Pathways related to CDSs corresponding to BACs:

1. path:sbi00052 - Galactose metabolism
2. path:sbi01230 - Biosynthesis of amino acids
3. path:sbi03013 - RNA transport
4. path:sbi00280 - Valine, leucine and isoleucine degradation
5. path:sbi04144 - Endocytosis

6. path:sbi03040 - Spliceosome
7. path:sbi00561 - Glycerolipid metabolism
8. path:sbi00790 - Folate biosynthesis
9. path:sbi00900 - Terpenoid backbone biosynthesis
10. path:sbi00240 - Pyrimidine metabolism
11. path:sbi04141 - Protein processing in endoplasmic reticulum
12. path:sbi03010 - Ribosome
13. path:sbi00040 - Pentose and glucuronate interconversions
14. path:sbi01212 - Fatty acid metabolism
15. path:sbi03008 - Ribosome biogenesis in eukaryotes
16. path:sbi00190 - Oxidative phosphorylation
17. path:sbi00564 - Glycerophospholipid metabolism
18. path:sbi00860 - Porphyrin and chlorophyll metabolism
19. path:sbi04070 - Phosphatidylinositol signaling system
20. path:sbi04145 - Phagosome
21. path:sbi00500 - Starch and sucrose metabolism
22. path:sbi00250 - Alanine, aspartate and glutamate metabolism
23. path:sbi03020 - RNA polymerase
24. path:sbi00960 - Tropane, piperidine and pyridine alkaloid biosynthesis
25. path:sbi01100 - Metabolic pathways
26. path:sbi00400 - Phenylalanine, tyrosine and tryptophan biosynthesis
27. path:sbi00051 - Fructose and mannose metabolism
28. path:sbi04016 - MAPK signaling pathway - plant
29. path:sbi00780 - Biotin metabolism
30. path:sbi04146 - Peroxisome
31. path:sbi00061 - Fatty acid biosynthesis
32. path:sbi01110 - Biosynthesis of secondary metabolites
33. path:sbi00970 - Aminoacyl-tRNA biosynthesis
34. path:sbi03440 - Homologous recombination
35. path:sbi03018 - RNA degradation
36. path:sbi00230 - Purine metabolism
37. path:sbi00591 - Linoleic acid metabolism
38. path:sbi00270 - Cysteine and methionine metabolism
39. path:sbi00260 - Glycine, serine and threonine metabolism
40. path:sbi00010 - Glycolysis/Gluconeogenesis
41. path:sbi04626 - Plant-pathogen interaction

Pathways related to CDSs corresponding to scaffolds found in the intersection analysis:

1. path:sbi03010 - Ribosome
2. path:sbi00902 - Monoterpenoid biosynthesis
3. path:sbi00051 - Fructose and mannose metabolism
4. path:sbi00052 - Galactose metabolism
5. path:sbi00940 - Phenylpropanoid biosynthesis

6. path:sbi01230 - Biosynthesis of amino acids
7. path:sbi00030 - Pentose phosphate pathway
8. path:sbi00920 - Sulfur metabolism
9. path:sbi03013 - RNA transport
10. path:sbi00511 - Other glycan degradation
11. path:sbi00062 - Fatty acid elongation
12. path:sbi03022 - Basal transcription factors
13. path:sbi01110 - Biosynthesis of secondary metabolites
14. path:sbi00250 - Alanine, aspartate and glutamate metabolism
15. path:sbi00500 - Starch and sucrose metabolism
16. path:sbi00564 - Glycerophospholipid metabolism
17. path:sbi03040 - Spliceosome
18. path:sbi00970 - Aminoacyl-tRNA biosynthesis
19. path:sbi04120 - Ubiquitin mediated proteolysis
20. path:sbi03018 - RNA degradation
21. path:sbi00513 - Various types of N-glycan biosynthesis
22. path:sbi00230 - Purine metabolism
23. path:sbi00380 - Tryptophan metabolism
24. path:sbi00600 - Sphingolipid metabolism
25. path:sbi01200 - Carbon metabolism
26. path:sbi00510 - N-Glycan biosynthesis
27. path:sbi00010 - Glycolysis/Gluconeogenesis
28. path:sbi01100 - Metabolic pathways
29. path:sbi04626 - Plant-pathogen interaction
